# Metallomic Signatures of Lung Cancer and Chronic Obstructive Pulmonary Disease

**DOI:** 10.3390/ijms241814250

**Published:** 2023-09-18

**Authors:** Belén Callejón-Leblic, Saida Sánchez Espirilla, Carolina Gotera-Rivera, Rafael Santana, Isabel Díaz-Olivares, José M. Marín, Ciro Casanova Macario, Borja García Cosio, Antonia Fuster, Ingrid Solanes García, Juan P. de-Torres, Nuria Feu Collado, Carlos Cabrera Lopez, Carlos Amado Diago, Amparo Romero Plaza, Luis Alejandro Padrón Fraysse, Eduardo Márquez Martín, Margarita Marín Royo, Eva Balcells Vilarnau, Antonia Llunell Casanovas, Cristina Martínez González, Juan Bautista Galdíz Iturri, Celia Lacárcel Bautista, José Luis Gómez-Ariza, Antonio Pereira-Vega, Luis Seijo, José Luis López-Campos, Germán Peces-Barba, Tamara García-Barrera

**Affiliations:** 1 Department of Chemistry, Research Center for Natural Resources, Health and the Environment (RENSMA), Faculty of Experimental Sciences, University of Huelva, Campus El Carmen, Fuerzas Armadas Ave., 21007 Huelva, Spain; belen.callejon@dqcm.uhu.es (B.C.-L.); saidik.s.05@gmail.com (S.S.E.); ariza@uhu.es (J.L.G.-A.); 2 Department of Chemistry, Faculty of Sciences, National University of San Antonio Abad of Cusco, Av. de La Cultura, Cusco 773, Peru; 3 IIS-Jiménez Díaz Foundation, ISCIII-CIBERES, Reyes Católicos Ave., 28040 Madrid, Spain; carolina.gotera@fjd.es (C.G.-R.); rafael.santana@quironsalud.es (R.S.); 4 Beturia Andalusian Foundation for Health Research (FABIS), Ronda Norte, s/n, 21005 Huelva, Spain; fabis_ne.tai.hujrj@fabis.org; 5 Miguel Servet Hospital-IIS Aragon, ISCIII-CIBERES, Paseo de Isabel la Católica, 1-3, 50009 Zaragoza, Spain; jmmarint@unizar.es; 6 Pulmonary Department—Research Unit, Hospital Universitario Nuestra Señora de Candelaria, CIBERES, ISCIII, Universidad de La Laguna, Padre Herrera, s/n, 38200 Santa Cruz de Tenerife, Spain; casanovaciro@gmail.com; 7 Son Espases Hospital, IdISBa, ISCIII-CIBERES, Valldemossa Road, 79, 07120 Palma De Mallorca, Spain; borja.cosio@ssib.es; 8 Son Llàtzer Hospital, C. de Manacor, 07198 Palma, Spain; afuster@hsll.es; 9 Santa Creu i Sant Pau Hospital, Carrer de St. Antoni Maria Claret, 167, 08025 Barcelona, Spain; isolanes@santpau.cat; 10 University Clinic of Navarra, Pío XII Ave., 36, 31008 Pamplona, Spain; jupa65@hotmail.com; 11 Reina Sofía Hospital, Maimonides Institute for Biomedical Research of Córdoba, Menéndez Pidal Ave., s/n, 14004 Córdoba, Spain; nurifeco@yahoo.es; 12 University Hospital of Gran Canaria Dr. Negrín, Respiratory Service, C. Pl. Barranco de la Ballena, s/n, 35010 Las Palmas de Gran Canarias, Spain; ccablopn@gmail.com; 13 Valdecilla Hospital, Valdecilla, s/n, 39008 Santander, Spain; amadodiago.carlos@gmail.com; 14 Manacor Hospital, Manacor Alcudia Road, s/n, 07500 Manacor, Spain; aromero@hmanacor.org; 15 Pneumology Area of the Juan Ramón Jiménez Hospital, Ronda Norte, s/n, 21005 Huelva, Spain; luispadronf@gmail.com (L.A.P.F.); apv01h@gmail.com (A.P.-V.); 16 Virgen del Rocío Hospital, Institute of Biomedicine of Seville (IBiS), ISCIII-CIBERES, Manuel Siurot Ave., s/n, 41013 Seville, Spain; eduardomarquezmartin@gmail.com; 17 Castellón Hospital, Benicàssim Ave., 128, 12004 Castellón, Spain; marin_marroy@gva.es; 18 Hospital del Mar, ISCIII-CIBERES, Paseo Marítimo de la Barceloneta, 25, 29, 08003 Barcelona, Spain; ebalcells@parcdesalutmar.cat; 19 Terrassa Hospital, Torrebonica Road, s/n, 08227 Barcelona, Spain; allunell@cst.cat; 20 Central Hospital of Asturias, Roma Ave., s/n, 33011 Oviedo, Spain; 21 Cruces University Hospital, ISCIII-CIBERES, Cruces Plaza, s/n, 48903 Baracaldo, Spain; med001901@hotmail.com; 22 Jaén University Hospital, Av. del Ejército Español, 10, 23007 Jaén, Spain; celaba74@gmail.com; 23 University Clinic of Navarra, ISCIII-CIBERES, Monforte de Lemos Ave., 28029 Madrid, Spain; lseijo@unav.es; 24 Medical-Surgical Unit for Respiratory Diseases, Institute of Biomedicine of Seville (IBiS), Virgen del Rocío University Hospital, University of Seville, Manuel Siurot Ave., s/n, 41013 Sevilla, Spain; lopezcampos@us.es; 25 Center for Biomedical Research in Respiratory Diseases Network (CIBERES), Carlos III Health Institute, Av. de Monforte de Lemos, 3–5, 28029 Madrid, Spain

**Keywords:** lung cancer, chronic obstructive pulmonary disease, metals, inductively coupled plasma, metallomics, mass spectrometry

## Abstract

Lung cancer (LC) is the leading cause of cancer deaths, and chronic obstructive pulmonary disease (COPD) can increase LC risk. Metallomics may provide insights into both of these tobacco-related diseases and their shared etiology. We conducted an observational study of 191 human serum samples, including those of healthy controls, LC patients, COPD patients, and patients with both COPD and LC. We found 18 elements (V, Al, As, Mn, Co, Cu, Zn, Cd, Se, W, Mo, Sb, Pb, Tl, Cr, Mg, Ni, and U) in these samples. In addition, we evaluated the elemental profiles of COPD cases of varying severity. The ratios and associations between the elements were also studied as possible signatures of the diseases. COPD severity and LC have a significant impact on the elemental composition of human serum. The severity of COPD was found to reduce the serum concentrations of As, Cd, and Tl and increased the serum concentrations of Mn and Sb compared with healthy control samples, while LC was found to increase Al, As, Mn, and Pb concentrations. This study provides new insights into the effects of LC and COPD on the human serum elemental profile that will pave the way for the potential use of elements as biomarkers for diagnosis and prognosis. It also sheds light on the potential link between the two diseases, i.e., the evolution of COPD to LC.

## 1. Introduction

Lung cancer (LC) is the most common cause of cancer deaths and has led to more than 1.8 million deaths and 2.1 million new cases in 2020 [1]. Chronic obstructive pulmonary disease (COPD) is the third leading cause of death worldwide [2], and it is known to increase the risk of LC. COPD is therefore the foremost public health problem today, and this problem is expected to worsen in the near future. Elements are essential because about one third of human proteins need them in order to develop and perform their functions [3], and it has been suggested that their impairment is both a cause (due to the exposure to high levels) and an effect of LC onset and progression, which can lead to unbalanced levels. Metal ions can play important roles as “matchmakers” for more than 50% of known proteins [4], and it has been reported that the activity of antioxidant enzymes is linked to the concentration of elements [5]. In fact, it has been shown that 47% of structurally determined proteins require the presence of metals, which make up 41% of their catalytic centers. Moreover, the concentrations of particular elements in biofluids should not be investigated in isolation, since numerous synergistic and antagonistic interactions among different elements have been described [6]. Thus, elements may be altered during cancer progression [7,8,9] and metastasis [10,11], and elemental dyshomeostasis has been reported in LC human biofluids [12,13,14,15,16]. Some authors have investigated the elemental concentrations in serum samples from LC [15,17,18,19] or COPD [20,21,22] patients, but no previous study has considered samples from patients with COPD who have developed LC during follow-up. In addition, most published articles have focused on the relationship between metal exposure and these diseases instead of the potential associations between unbalanced levels and both LC and COPD.

The aim of this work was to assess the effects of metals on both LC and COPD independently, as well as to evaluate the serum elemental profiles of COPD patients who developed LC during follow-up (COPD-LC), to establish whether there is an association between the two diseases. For this reason, we analyzed the human serum elemental profiles of LC, COPD, and COPD-LC patients via inductively coupled plasma mass spectrometry (ICP-MS). The elemental profiles of a group of healthy control (HC) patients were also determined in order to compare them with those of the other groups. In addition, we evaluated differences in the elemental compositions of COPD patients whose disease severity varied from mild to very severe. This study delves into the impact LC and COPD on the human serum elemental profile.

## 2. Results

### 2.1. The Human Serum Elemental Profile Is Influenced by LC and COPD

**Figure 1:**
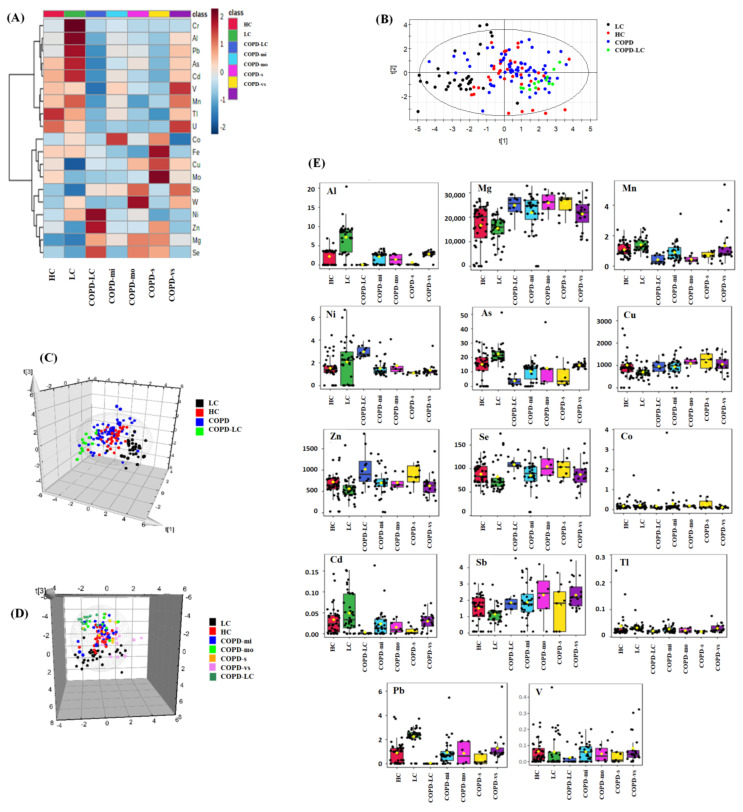
Impact of LC and COPD on the human serum metallomic profile. (**A**) Heatmap showing the serum elemental profiles of the different groups under study (*p* < 0.05) (adjusted by FDR correction). (**B**) Principal component analysis (PCA). (**C**) Scatter plot of partial least squares-discriminant analysis (PLS−DA). Classification of HC, LC, COPD and COPD-LC groups based on their elemental profile of human serum. (**D**) Scatter plot of partial least squares-discriminant analysis (PLS-DA). Classifications of HC, LC, COPD of varying severity (COPD−mi, COPD−mo, COPD−s, and COPD−vs), and COPD−LC groups based on their serum elemental profiles. (**E**) Box plots of levels determined for the most significantly altered elements in the serum samples of the groups under study.

**Figure 2:**
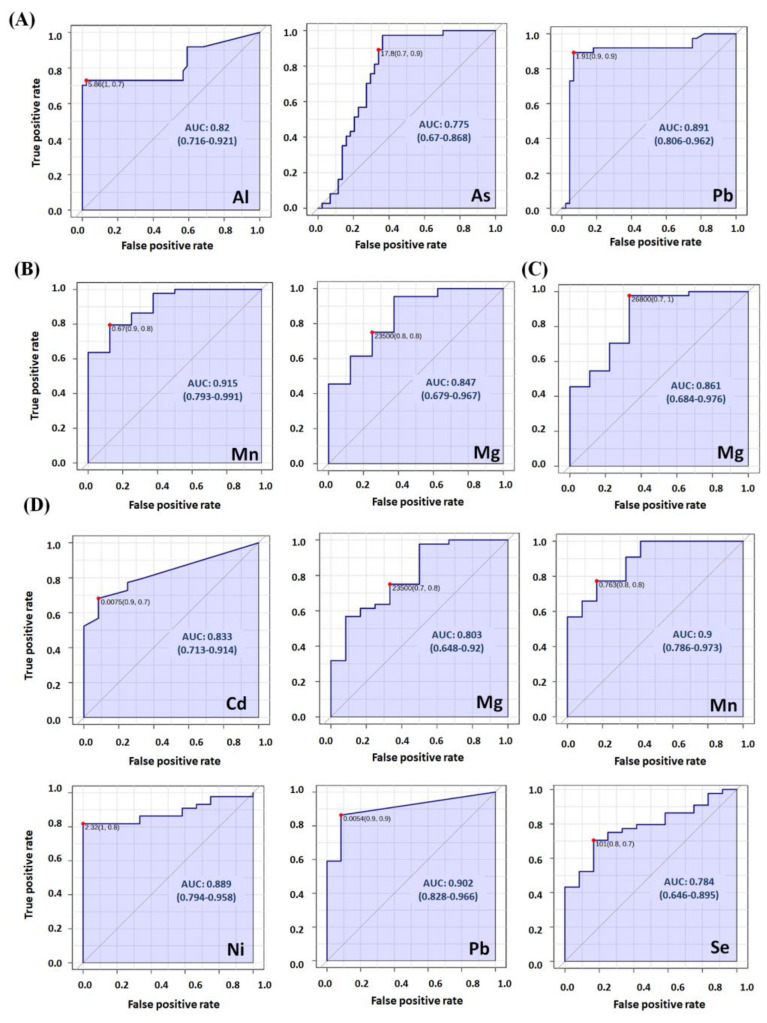
ROC curves showing significant differences between elements in the (**A**) LC, (**B**) COPD-mo, (**C**) COPD-s, and (**D**) COPD-LC groups compared with those in the HC group (AUC values higher than 0.75).

The average serum elemental concentrations (Supplementary Table S1), fold changes, *p*-values, and AUC values from ROC curves (Supplementary Table S2) of the studied groups are collected in the Supplementary Material. Compared with those of the HCs (Figure 1A, Supplementary Table S2), the human serum metallomic profiles of the LC patients showed significantly increased concentrations of Al (2.35-fold, *p* = 0.00), As (1.35-fold, *p* = 0.00), Mn (1.26-fold, *p* = 0.00), and Pb (1.87-fold, *p* = 0.00), while the COPD patients had increased levels of Mg (1.38-fold, *p* = 0.00) and Sb (1.33-fold, *p* = 0.01) and reduced levels of As (0.68-fold, *p* = 0.00), Cd (0.59-fold, *p* = 0.02), and Tl (0.67-fold, *p* = 0.04). Interestingly, the COPD-LC group presented the most significantly altered metallomic profiles when compared with the HC group, with increased levels of Mg (1.60-fold, *p* = 0.00), Ni (1.37-fold, *p* = 0.00), and Se (1.33-fold, *p* = 0.00) and reduced concentrations of Al (0.02-fold, *p* = 0.00), As (0.23-fold, *p* = 0.00), Cd (0.08-fold, *p* = 0.01), Mn (0.41-fold, *p* = 0.00), and Pb (0.04-fold, *p* = 0.00). The metallomic profiles were also different when comparing HC, LC, and COPD-LC patients against COPD patients with varying disease severity (from mild to very severe symptoms). However, a comparison among the COPD patients with different disease severities revealed that the metallomic profiles differed only between COPD-vs vs. COPD-mo and COPD-vs vs. COPD-se. Thus, no significant differences were observed between COPD-mo vs. COPD-mi, COPD-se vs. COPD-mi, COPD-vs vs. COPD-mi, or COPD-s vs. COPD-mo. A principal component analysis (PCA) based on the metallomic profiles enabled us to cluster the serum samples of the HC, LC, COPD, and COPD-LC patients (Figure 1B). A PLS-DA plot also confirmed the clear separation between the groups (Figure 1C). Moreover, a PLS-DA plot which includes the COPD patients with varying disease severity (Figure 1D) is shown in Figure 1. Other PLS-DA plots which include two or three groups are shown in Supplementary Figure S1 and enable a better visualization. Variable importance in projection (VIP) is a parameter that indicates the most affected variables in the PLS-DA model. VIPs higher than 1 suggest significant differences between groups in the multivariate analysis. The PLS-DA showed VIPs higher than 1 for Al, As, Pb, and Zn in several comparisons, indicating that these are the most affected elements with respect to the rest of the metals. The most affected elements in the different PLS-DAs were as follows: Al (VIP = 2.06) and Pb (VIP = 2.06) in the comparison between LC and HC; Al (VIP = 1.89) and As (VIP = 1.60) in the comparison between COPD and LC; Ni (VIP = 2.64) in the comparison between COPD and COPD-LC; and Cd (VIP = 2.04), Mn (VIP = 1.68), Al (VIP = 1.48), and Sb (VIP = 1.53) in the comparisons between COPD-mi, COPD-mo, COPD-s, and COPD-vs. Supplementary Table S3 shows the VIP values for the other metal group comparisons. The different concentrations of the most altered elements in the serum samples of the studied groups are also shown in the box plots (Figure 1E). Figure 2 shows the ROC curves that correspond to the elements whose concentrations differed significantly between the LC (Figure 2A), COPD-mo (Figure 2B), COPD-s (Figure 2C), and COPD-LC (Figure 2D) groups and the HC group. Elements with AUC values higher than 0.75 could have clinical utility as possible biomarkers [23]. AUC values are reported in Supplementary Table S2. Since As, Al, Mn, Pb, Mg, and Cr presented AUC values > 0.75 (Supplementary Figure S2), these could function as possible biomarkers for LC vs. COPD-LC (Supplementary Figure S2A). As could function as possible biomarker for LC vs. COPD-mi (Supplementary Figure S2B), As, Cr, and Sb could function as possible biomarkers for LC vs. COPD-vs (Supplementary Figure S2C), and Ni and Pb could function as possible biomarkers for COPD-LC vs. COPD-mi (Supplementary Figure S2D).

### 2.2. Altered Elemental Ratios Influenced by LC and COPD

As was previously mentioned, the elemental dyshomeostasis has been reported to be related to lung cancer. Moreover, synergistic and antagonistic interactions between elements have been extensively described. For this reason, the ratios between the concentrations of elements in the serum samples were also evaluated as possible signatures of LC, COPD, and the other subgroups under study (Supplementary Tables S4–S7). In general, the pairwise comparisons among the studied groups presented many elemental ratios that were significantly different, and which had significant FC values.

We mainly found significantly increased ratios of Al to the oligoelements Co, Cu, Fe, Mn, Mo, Se, and Zn in the LC group compared with the HC and COPD groups (Supplementary Tables S4 and S5), as well significantly increased ratios of Mg to Mn and Zn in the COPD group compared with the HC group (Supplementary Table S5). Moreover, significant ratios of Mn to Se and Zn were observed in the comparison between the COPD-LC and COPD groups (Supplementary Table S6). Only a few significantly different ratios were found when comparing the groups with COPD of varying severity (COPD-mi, COPD-mo, and COPD-s) (Supplementary Table S7). The specificity and sensitivity of the metal ratios were also evaluated by analyzing the ROC curves. Supplementary Table S8 shows the ratios which had AUC values higher than 0.75 in the different groups following comparisons with the controls. The ratios between Al and the majority of the analyzed elements presented higher and better AUC values in the comparison between the LC and HC groups. In addition, the AUC values of some of the ratios of Mg, Mn, and Ni to the rest of the elements in the COPD-LC group were found to be higher than those of the control group (Supplementary Table S8). In contrast, no good AUC values were observed in the COPD group.

### 2.3. Associations between Metals in COPD (of Varying Severity) and LC

**Figure 3:**
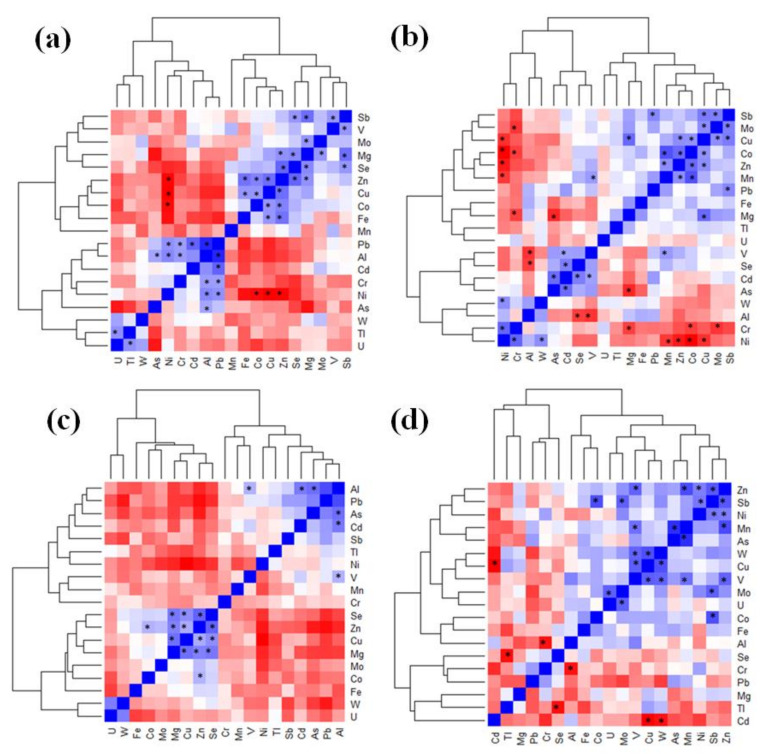
Spearman correlation heatmap showing correlations between metals in the (**a**) HC, (**b**) LC, (**c**) COPD, and (**d**) COPD-LC groups. * significant (*p* < 0.05) and strong (rho > 0.5) correlations.

A Spearman correlation analysis was applied to the dataset to determine possible associations between metals in the different studied groups. Significant (*p* < 0.05) and strong (rho > 0.5) (Supplementary Table S9) correlations were calculated and are presented in the Supplementary Material. After pairwise compassions between the levels of all the elements in the serum samples, some of them were found to be negatively (rho < 0) or positively (rho > 0) associated; thus, as the concentration of one element increased, the concentration of the other decreased just as much, or vice versa. In addition, in some cases, the significant associations were lineal (rho ≈ ±1). We found a higher number of metal–metal associations in the HC (Figure 3a) and LC groups (Figure 3b). In the control group, Zn were positively correlated with several oligoelements, such as Se (rho = 0.69, *p* < 0.05), Cu (rho = 0.66, *p* < 0.05), Fe (rho = 0.65, *p* < 0.05), Co (rho = 0.60, *p* < 0.05), and Mg (rho = 0.56, *p* < 0.05). These correlations were not significant in the LC and COPD-LC groups. In contrast, Zn was positively correlated with Se (rho = 0.73, *p* < 0.05), Mg (rho = 0.65, *p* < 0.05), and Cu (rho = 0.54, *p* < 0.05) in the COPD group (Figure 3c). The levels of Cu and Ni were correlated with those of several metals in the LC patients. Cu was positively correlated with Mg (rho = 0.65, *p* < 0.05), Sb (rho = 0.57, *p* < 0.05), Zn (rho = 0.53, *p* < 0.05), and Co (rho = 0.52, *p* < 0.05). Ni was positively associated with Cr (rho = 0.64, *p* < 0.05) and W (rho = 0.53, *p* < 0.05), and it was negatively associated with Mn (rho = −0.55, *p* < 0.05), Zn (rho = 0.64, *p* < 0.05), and Co (rho = −0.73, *p* < 0.05). Moreover, we found positive associations between V and Cu (rho = 0.68, *p* < 0.05), W (rho = 0.67, *p* < 0.05), Zn (rho = 0.66, *p* < 0.05), and Mn (rho = 0.63, *p* < 0.05) in the COPD-LC group (Figure 3d).

## 3. Discussion

Our study suggests that LC, COPD, COPD-LC, and COPD of varying severity have a strong impact on the metallomic profile of human serum. In general, we can distinguish two groups of elements, namely toxic (As, Cd, Sb, Pb, Tl, Cr, Ni, and U) and bioelements (V, Al, Mn, Co, Cu, Zn, Se, Mo, and Mg) [24]. No specific health effects have been associated with exposure to W in humans, but it is not a bioelement [25].

The metallomic profiles of the serum samples indicated significantly higher levels of several carcinogenic elements, such as As and Ni, in the LC group compared with the HC, COPD, and COPD-LC groups. Exposure to these elements is known to be hazardous [26] because they can activate oncogenic signaling pathways [27,28] and induce oxidative stress [26,29,30]. Ni is a pulmonary carcinogen that induces oxidative DNA damage [31], while As and Pb exposure is associated with decreased lung function [21]. Furthermore, Cd, which is abundant in cigarette smoke, can destroy lung tissue, leading to chronic bronchitis and emphysema [32]. Interestingly, we found lower levels of Cd and As in the COPD patients than in the HC patients, although the levels were significantly higher in patients with very severe COPD than in those with milder forms of the disease. It is well known that tobacco smoke, in which heavy metals are present, is significantly associated with an increased incidence of smoking-related COPD, and that it can accelerate physiological processes, leading to oxidative stress, damage, and inflammation [33]. However, some authors have concluded that smoking cessation treatment slows the decline in respiratory function and leads to clinical–functional improvements [34,35].

A redox imbalance is critical to lung carcinogenesis and has been associated with alterations in the serum concentrations of Zn, Mn, and Cu. These metals play a key role in antioxidant activity (mainly enzymatic processes) as cofactors or ions which stabilize the molecular structure of superoxide dismutase (SOD), an endogenous antioxidant [36]. Mn-SOD, which is of key importance in the lungs’ antioxidant defense [37], is the only superoxide radical scavenger in mitochondria and therefore precludes antioxidant and tumor-suppressor cell function [38]. Cu is an essential trace element, but it also contributes to the generation of free radicals [13], while Zn is a constituent of over 300 enzymes that play key roles in gene expression [39].

The mechanisms by which specific metallomic profiles are linked to disease states are unclear as imbalances in the latter can lead to complex interactions through competitive, antagonistic, or synergistic mechanisms [6]. For this reason, we analyzed not only metallomic profiles, but also the specific ratios between elements, thereby identifying significant differences among the studied groups. Most of these elements were also altered in our previous study, which was carried out on different biofluids of LC patients (though with a smaller cohort) [15]. On the other hand, the significant alterations in the levels of Al in the LC patients and the COPD patients with LC might be due to imbalances in energy metabolism since citrates have been identified as one of the main molecules which interact with this metal [40], and as playing a central role in cancer cell metabolism and regulation [41]. On the other hand, Al is considered a potential endocrine disruptor, and human exposure has been associated with increased lung cancer risk [42].

The increased levels of Mg in the COPD patients compared with the HC could be associated with possible alterations in protein synthesis and bone metabolism [20]. Some authors have suggested that Mg works as a bronchodilator by inhibiting calcium influx into the smooth muscles of the bronchioles [43]. However, Mg’s role in obstructive lung diseases is unclear [44].

Se is one of the most studied elements in the metallomic profiling of LC due to its antioxidant properties, and because it is a cofactor of selenoproteins, which are essential antioxidants [45]. However, we found no significant differences in the total concentrations of Se, with the exception of the COPD patients with LC; this warrants further study as Se concentration may be a biomarker of the two tobacco-related diseases and could help explain whether COPD is an independent risk factor for LC.

It is generally accepted that AUC values above 0.75 indicate useful biomarkers with potential clinical applications by ensuring a reasonable combination of sensitivity and specificity [23]. In this sense, our study could be viewed as a first step in the identification of metal concentrations or metal ratios that may work as potential biomarkers of LC and COPD, or as harbingers of LC in susceptible COPD patients. Both Al and Mn, which had AUC values close to 1, appear to be of special interest in our study.

Finally, differences in the associations between metals in the LC, COPD, and COPD-LC patients compared with the HC patients could indicate dysregulation in metal homeostasis. It is well known that there are many types of biological processes that are modulated by the availability of metals, and that these can also contribute to the pathogenesis of many different types of cancer-preventing or -accelerating neoplastic cell transformations, and to the modulating of the inflammatory and pro-tumorigenic response in immune cells [46].

## 4. Study Limitations

The number of serum samples from the COPD-LC patients (*n* = 16) was small compared with those from the HC (*n* = 56), COPD (*n* = 74), and LC (*n* = 47) patients. A larger population of COPD-LC patients would be necessary to validate the results. Similarly, the number of serum samples from the COPD subgroups COPD-mo (*n* = 10) and COPD-s (*n* = 10) were smaller than those of the other groups, COPD-mi (*n* = 33) and COPD-vs (*n* = 21).

## 5. Materials and Methods

### 5.1. Study Participants and Study Design

We determined the concentrations of 18 elements in 191 serum samples from 47 LC and 90 COPD patients that were compared with samples from 54 healthy controls (HCs). The COPD group was also divided in several subgroups according to disease severity, based on pulmonary function testing [47], as follows: very severe COPD (COPD-vs, *n* = 21), severe COPD (COPD-s, *n* = 10), moderate COPD (COPD-mo, *n* = 10), mild COPD (COPD-mi, *n* = 33), and COPD patients who developed LC during follow-up (COPD-LC, *n* = 16).

COPD History Assessment in SpaiN (CHAIN) is a multicenter, observational study of prospective cohorts which is being carried out at 36 Spanish hospitals. The recruitment period began on 15 January 2010 and is ongoing (clinicaltrials.gov identifier: NCT01122758). All participants have signed informed consent forms which have been approved by the ethics committees of the participating centers (Hospital Universitario la Candelaria, Tenerife, Spain; IRB No. 258/2009). The cohort is active and has a follow-up period of more than 10 years, with complete office visits every 12 months and telephone interviews every 6 months, in order to evaluate exacerbations and monitor the vital state of each subject. The data analyzed in this study were collected from the date of recruitment until May 2015. The data were anonymized with hierarchical access control to ensure data security. At each annual visit, the following information was collected: (i) clinical aspects (socio-economic situation, anthropometric data, comorbidities, smoking, respiratory symptoms, exacerbations, quality of life, anxiety–depression scale, daily life activities, treatments); (ii) respiratory function (spirometry, blood gases, hyperinflation, diffusion, respiratory pressures); (iii) BODE index (main study variable); (iv) peripheral muscle function; and (v) blood work-up (including IgE and cardiovascular risk factors). In addition, a serum bank was created for the future determination of biomarkers, and some of the samples analyzed in this study were taken from this. The rest of the samples were collected at the pneumology area of the Juan Ramón Jiménez Hospital of Huelva, Spain during 2018–2020.

The blood samples were obtained by venipuncture of the antecubital region using BD Vacutainer SST II tubes with gel separator and Advance vacuum system. The samples were then cooled and protected from light to prevent clotting (t = 30 min). After centrifugation (2000 g, 10 min), the serum samples were frozen at −80 °C until analysis. The study was performed in accordance with the principles contained in the Declaration of Helsinki and approved by the Ethics Committee of the regional Andalusian Government (Ethical code num. 1898-N-21).

Patient data were anonymized in a database with hierarchical access control in order to guarantee secure information access. Clinical parameters such as age, gender, LC or COPD stage, smoking habits, and comorbidities are shown in Table 1.

### 5.2. Determination of Elements in Serum Samples via ICP-MS

For simple and fast sample preparation, a previously reported method was used [48]. Briefly, the serum samples (0.5000 g) were weighted and diluted with 4.5 mL of a solution containing 0.05% ethylenediaminetetraacetic acid (EDTA, 99.995% trace metals basis), 0.05% triton-X 100, 5% 1-butanol, and 0.25% NH4OH. Rh, Sc, and Bi were added as internal standards (100 ng g^−1^ each).

A multielement analysis was carried out in a triple quadrupole ICP-MS model 8800 (Agilent Technologies, Tokyo, Japan) using collision gas (helium) at a flow rate of 4.5 mL min^−1^. A mixture of H_2_ (2 mL min^−1^) and O_2_ (40%) was used in the MS/MS mode for Se. The following isotopes were monitored (0.3 s of dwell time per isotope): ^24^Mg, ^27^Al, ^51^V, ^55^Mn, ^57^Fe, ^59^Co, ^53^Cr, ^60^Ni, ^63^Cu, 65Cu, ^64^Zn, ^66^Zn, ^75^As, ^78^Se, ^80^Se, ^95^Mo, ^103^Rh, ^112^Cd, ^114^Cd, ^182^W,^205^Tl, ^208^Pb, and ^238^U. A tuning aqueous solution of Li, Co, Y, and Tl at 1 μg L^−1^ was used to tune the ICP-MS.

A multielement calibration standard-2A solution (10 mg L^−1^ of Al, V, Cr, Mn, Fe, Co, Ni, Cu, Zn, As, Se, Cd, Tl, and Pb) (Agilent Technologies) was used to prepare multielemental internal calibrations curves. After reshaping the calibration function, the concentrations at the detection (LOD) and quantification (LOQ) limits were obtained [1], and elemental levels below the LOQ were excluded from further statistical evaluations. The samples were performed five times for the instruments (*n* = 5). Supplementary Table S10 shows the LODs and LOQs for the elements.

The validation of the multielemental methodology was carried out using two serum reference materials: Seronorm trace elements reference material at level L-2 (SERO AS, Hvalstad, Norway) and ClinChek, serum control, lyophilized, for trace element analysis, level II (RECIPE, München, Germany). As is shown in Supplementary Table S11, the results for accuracy and precision (related standard deviation, RSD) obtained in the internal quality control procedure were within the certified ranges.

Other operational parameters for ICP-MS, such as sampling depth, RF forward power, carried gas flow rate, or type of nebulizer, are detailed in Supplementary Table S12.

### 5.3. Statistics

Statistica 8 (Statsoft, Tulsa, OK, USA) was used to analyze the data. The concentrations of elements and selenoproteins in the serum samples were expressed as medians and standard errors of the means (S.E.) (median ± S.E.). A Levens’s test revealed no homogenous variances or skewed distributions (checked using normal probability plots), and all the data were analyzed using nonparametric methods. Group comparisons were performed using Krustal–Wallis tests, and only *p*-values < 0.05 were considered statistically significant. When possible, fold changes (FCs) were calculated for pairwise comparisons among the studied groups by dividing the median concentrations of elements determined in the serum samples. The areas under the receiver operating characteristic curves (AUC values from ROC curves) were also calculated so that we could delve into the specificity and sensitivity of the novel potential biomarkers. In addition, partial least squares-discriminant analyses (PLS-DAs) were performed and heatmaps were produced to investigate the potential contributions of the elemental compositions present in the serum samples and discriminate the studied groups. MetaboAnalyst version 5.0 was used as a statistical tool for the ROC curves and heatmaps “https://www.metaboanalyst.ca/ (accessed on 15 December 2022)”.

## 6. Conclusions

In this work, we determined the elemental concentrations in serum samples from LC subjects and COPD patients with varying disease severity, including a group of patients who developed LC during follow-up, using ICP-MS. Our results indicate significant alterations in the concentrations of several elements, including Al, As, Pb, Mg, Mn, Cd, Ni, and Se in the LC, COPD, and COPD-LC groups compared with the controls, and this could be of clinical utility due to the good AUC values obtained from the ROC analysis. We found significant changes in some elements, especially in the LC and COPD-LC groups compared with the HC group. Furthermore, we reported numerous associations between elements, especially in the LC group, indicating intertwined mechanisms and dyshomeostasis, both of which should be further explored.

## Figures and Tables

**Table 1:** Clinical characteristics of patients included in the study.

Clinical Characteristics		HC	LC	COPD	COPD-LC
Gender (male/female)		38/16	29/18	60/14	15/1
Age (years ± SD)		61 ± 11	66 ± 11	65 ± 9	56 ± 8
Smoking habits	Non-smoker	24	11	0	1
	Smoker	15	24	42	6
	Ex-smoker	15	12	32	9
	Adenocarcinoma	-	33	-	4
NSCLC type	Carcinoide	-	2	-	6
	Squamous cell lung cancer	-	8	-	2
	Epidermoide	-	4	-	4
	Mild	-	-	33	4
COPD grading	Moderate	-	-	10	8
	Severe	-	-	10	0
	Very Severe	-	-	21	4
Comorbidities	DM	5	8	7	3
	AH	17	18	34	11
	DLP	20	25	21	7

HC: healthy control; LC: lung cancer; COPD: chronic obstructive pulmonary disease; COPD-LC: COPD patients who developed LC during follow-up: COPD-vs: very severe COPD; COPD-s: severe COPD; COPD-mo: moderate COPD; COPD-mi: mild COPD; DM: diabetes mellitus; AH: arterial hypertension; DLP: dyslipidemia.

## Data Availability

Data are available on request.

## Supplementary Materials

The following supporting information can be downloaded at: https://www.mdpi.com/article/10.3390/ijms241814250/s1.

## Abbreviations

AF	Affinity chromatography
AUC	Area under the curve
COPD	Chronic obstructive pulmonary disease
COPD-LC	COPD patients who developed LC
COPD-vs	Very severe COPD
COPD-s	Severe COPD
COPD-mo	Moderate COPD
COPD-mi	Mild COPD
CHAiN	COPD History Assessment in SpaiN
HC	Healthy control
ICP-MS	Inductively coupled plasma mass spectrometry
LC	Lung cancer
LOD	Limits of detection
LOQ	Limits of quantification
PCA	Principal component analysis
PLS-DA	Partial least squares-discriminant analysis
ROC	Receiver operating characteristic
VIP	Variable importance in projection
