# Supplementary material for: Metallomic Signatures of Lung Cancer and Chronic Obstructive Pulmonary Disease
Source: Int J Mol Sci. 2023 Sep 18;24(18):14250. doi: 10.3390/ijms241814250 (PMC10532173; doi:10.3390/ijms241814250)
Supplement: Supplementary file 1 [file ijms-24-14250-s001.zip › ijms-2555996-supplementary.pdf]

Supplementary Material

Metallomic Signatures of Lung Cancer and Chronic Obstructive Pulmonary Disease

Belén Callejón-Leblic [1,†], Saida Sánchez Espirilla [1,2,†], Carolina Gotera-Rivera [3], Rafael Santana [3], Isabel Díaz-Olivares [4], José M. Marín [5], Ciro Casanova Macario [6], Borja García Cosio [7], Antonia Fuster [8], Ingrid Solanes García [9], Juan Pablo Torres [10], Nuria Feu Collado [11], Carlos Cabrera Lopez [12], Carlos Amado Diago [13], Amparo Romero Plaza [14], Luis Alejandro Padrón Fraysse [15], Eduardo Márquez Martín [16], Margarita Marín Royo [17], Eva Balcells Vilarnau [18], Antonia Llunell Casanovas [19], Cristina Martínez González [20], Juan Bautista Galdíz Iturri [21], Celia Lacárcel Bautista [22], José Luis Gómez-Ariza [1], Antonio Pereira-Vega [15], Luis Seijo [23], José Luis López-Campos [24,25], Germán Peces-Barba [3,*] and Tamara García-Barrera [1,*]

[1] Department of Chemistry, Research Center for Natural Resources, Health and the Environment (RENSMA), Faculty of Experimental Sciences, University of Huelva, Campus El Carmen, Fuerzas Armadas Ave., 21007 Huelva, Spain; belen.callejon@dqcm.uhu.es (B.C.-L.); saidik.s.05@gmail.com (S.S.E.); ariza@uhu.es (J.L.G.-A.).
[2] Department of Chemistry, Faculty of Sciences, National University of San Antonio Abad of Cusco, Av. de La Cultura, Cusco 773, Peru
[3] IIS-Jiménez Díaz Foundation, ISCIII-CIBERES, Reyes Católicos Ave., 28040 Madrid, Spain; carolina.gotera@fjd.es (C.G.-R.); rafael.santana@quironsalud.es (R.S.)
[4] Beturia Andalusian Foundation for Health Research (FABIS), Ronda Norte s/n, 21005 Huelva, Spain; fabis_ne.tai.hujrj@fabis.org
[5] Miguel Servet Hospital-IIS Aragon, ISCIII-CIBERES, Paseo de Isabel la Católica, 1-3, 50009 Zaragoza, Spain; jmmarint@unizar.es
[6] Pulmonary Department—Research Unit, Hospital Universitario Nuestra Señora de Candelaria, CIBERES, ISCIII, Universidad de La Laguna, Padre Herrera, s/n, 38200 Santa Cruz de Tenerife, Spain; casanovaciro@gmail.com
[7] Son Espases Hospital, IdISBa, ISCIII-CIBERES, Valldemossa Road, 79, 07120 Palma De Mallorca, Spain; borja.cosio@ssib.es
[8] Son Llàtzer Hospital, C. de Manacor, 07198 Palma, Spain; afuster@hsll.es
[9] Santa Creu i Sant Pau Hospital, Carrer de St. Antoni Maria Claret, 167, 08025 Barcelona, Spain; isolanes@santpau.cat
[10] University Clinic of Navarra, Pío XII Ave., 36, 31008 Pamplona, Spain; jupa65@hotmail.com
[11] Reina Sofía Hospital, Maimonides Institute for Biomedical Research of Córdoba, Menéndez Pidal Ave., s/n, 14004 Córdoba, Spain; nurifeco@yahoo.es
[12] University Hospital of Gran Canaria Dr. Negrín, Respiratory Service, C. Pl. Barranco de la Ballena, s/n, 35010 Las Palmas de Gran Canarias, Spain; ccablopn@gmail.com
[13] Valdecilla Hospital, Valdecilla, s/n, 39008 Santander, Spain; amadodiago.carlos@gmail.com
[14] Manacor Hospital, Manacor Alcudia Road, s/n, 07500 Manacor, Spain; aromero@hmanacor.org
[15] Pneumology Area of the Juan Ramón Jiménez Hospital, Ronda Norte, s/n, 21005 Huelva, Spain; luispadronf@gmail.com (L.A.P.F.); apv01h@gmail.com (A.P.-V.)
[16] Virgen del Rocío Hospital, Institute of Biomedicine of Seville (IBiS), ISCIII-CIBERES, Manuel Siurot Ave. s/n, 41013 Seville, Spain; eduardomarquezmartin@gmail.com
[17] Castellón Hospital, Benicàssim Ave., 128, 12004 Castellón, Spain; marin_marroy@gva.es
[18] Hospital del Mar, ISCIII-CIBERES, Paseo Marítimo de la Barceloneta, 25, 29, 08003 Barcelona, Spain; ebalcells@parcdesalutmar.cat

[19] Terrassa Hospital, Torrebonica Road, s/n, 08227 Barcelona, Spain; allunell@cst.cat
[20] Central Hospital of Asturias, Roma Ave. s/n, 33011 Oviedo, Spain; cmartinez@hca.es
[21] Cruces University Hospital, ISCIII-CIBERES, Cruces Plaza, s/n, 48903 Baracaldo, Spain; med001901@hotmail.com
[22] Jaén University Hospital, Av. del Ejército Español, 10, 23007 Jaén, Spain; celaba74@gmail.com
[23] University Clinic of Navarra, ISCIII-CIBERES, Monforte de Lemos Ave., 28029 Madrid, Spain; lseijo@unav.es
[24] Medical-Surgical Unit for Respiratory Diseases, Institute of Biomedicine of Seville (IBiS), Virgen del Rocío University Hospital, University of Seville, Manuel Siurot Ave. s/n, 41013 Sevilla, Spain; lopezcampos@us.es
[25] Center for Biomedical Research in Respiratory Diseases Network (CIBERES), Carlos III Health Institute, Av. de Monforte de Lemos, 3–5, 28029 Madrid, Spain
* Correspondence: gpeces@quironsalud.es (G.P.-B.); tamara@dqcm.uhu.es (T.G.-B.)
† These authors contributed equally to this work.

Table S1. Elemental concentration in human serum samples. Healthy Control (HC), Lung cancer (LC), Chronic Obstructive Pulmonary Disease (COPD), COPD patients who developed LC during the follow-up (COPD-LC), very severe COPD (COPD-vs), severe COPD (COPD-s), moderate COPD (COPD-mo) and mild COPD (COPD-mi); LOD: Limit of detection.

Groups	Parameters (ng g^{-1})	Mg	Al	V	Mn	Fe	Co	Ni	Cu	Zn	Cr	As	Mo	Se	Cd	Sb	W	Tl	Pb	U
HC	MEDIAN	15509.24	3.02	0.06	1.03	965.46	0.16	2.17	925.96	659.94	1.00	16.54	0.66	81.40	0.04	1.36	0.07	0.04	1.14	0.07
	SEM	1217.79	0.36	0.01	0.07	103.52	0.02	0.26	100.92	56.11	0.10	1.47	0.16	3.60	0.01	0.14	0.02	0.01	0.15	0.02
	MAX	27513.22	8.11	0.41	2.39	3004.93	0.74	7.95	3473.49	1862.14	3.64	52.43	6.23	134.90	0.18	4.11	0.84	0.29	3.87	0.79
	MIN	<LOD	<LOD	<LOD	0.40	11.01	0.01	<LOD	4.93	27.20	0.15	<LOD	<LOD	31.54	<LOD	<LOD	<LOD	0.01	<LOD	<LOD
LC	MEDIAN	14842.35	6.38	0.05	1.29	954.42	0.18	2.51	681.01	526.11	1.82	22.39	0.46	78.27	0.05	1.08	0.08	0.03	2.14	0.05
	SEM	744.61	0.42	0.01	0.07	86.47	0.04	0.27	38.96	33.01	0.54	0.89	0.06	4.84	0.01	0.08	0.01	0.00	0.11	0.01
	MAX	23819.81	9.54	0.46	2.50	3304.65	1.71	6.64	1447.83	1579.67	25.17	51.24	1.71	176.27	0.15	2.96	0.31	0.10	3.73	0.41
	MIN	2687.87	< LOD	<LOD	0.40	192.61	0.01	<LOD	160.85	141.64	0.62	13.02	<LOD	43.53	<LOD	<LOD	<LOD	0.01	0.01	<LOD
COPD	MEDIAN	22650.46	2.08	0.06	0.95	927.10	0.22	1.44	1024.99	701.17	0.68	11.49	0.61	89.72	0.02	2.03	0.08	0.03	0.95	0.05
	SEM	988.00	0.20	0.01	0.13	74.40	0.07	0.09	54.67	40.48	0.05	1.03	0.08	4.14	0.00	0.16	0.02	0.00	0.16	0.01
	MAX	32825.56	4.24	0.32	5.36	2595.21	3.84	4.07	1806.49	1438.55	3.38	44.53	3.73	153.16	0.16	4.44	0.80	0.08	6.38	0.52
	MIN	< LOD	<LOD	<LOD	<LOD	5.16	<LOD	0.81	2.13	12.00	0.27	<LOD	<LOD	<LOD	<LOD	<LOD	0.02	0.01	<LOD	0.01
COPD-LC	MEDIAN	24849.43	0.06	0.02	0.42	961.41	0.18	2.97	959.26	1017.81	0.62	3.78	0.54	108.41	0.01	1.86	0.05	0.02	0.05	0.02
	SEM	644.68	0.01	0.01	0.04	54.77	0.04	0.07	45.08	62.15	0.01	0.42	0.06	2.24	0.00	0.14	0.00	0.00	0.03	0.00
	MAX	29399.91	0.25	0.12	0.90	1656.91	0.98	3.93	1479.91	1851.67	0.71	8.76	1.31	140.22	0.02	4.58	0.07	0.04	0.59	0.03
	MIN	14659.82	< LOD	<LOD	0.11	380.60	0.04	2.39	535.35	468.37	0.53	0.35	<LOD	79.11	<LOD	0.46	0.03	0.01	<LOD	0.02
COPD-mi	MEDIAN	21914.01	2.14	0.06	0.90	874.89	0.29	1.51	937.78	699.94	0.73	10.40	0.55	84.72	0.02	1.89	0.05	0.03	0.94	0.03
	SEM	1150.35	0.21	0.01	0.10	64.01	0.10	0.10	55.90	44.62	0.07	0.89	0.04	4.36	0.00	0.16	0.01	0.00	0.16	0.00
	MAX	32825.56	4.24	0.20	3.44	1844.76	3.84	3.79	1806.49	1371.43	3.38	21.43	1.25	137.11	0.16	3.93	0.28	0.06	5.47	0.08
	MIN	< LOD	< LOD	<LOD	<LOD	5.16	<LOD	0.81	2.13	12.00	0.27	<LOD	<LOD	<LOD	<LOD	<LOD	0.02	0.01	<LOD	0.01
COPD-mo	MEDIAN	25859.84	1.43	0.05	0.44	822.19	0.20	1.74	1084.55	702.95	0.63	12.61	0.58	104.07	0.02	2.32	0.14	0.02	0.86	0.03
	SEM	629.20	0.21	0.01	0.03	52.09	0.02	0.14	38.34	25.05	0.01	2.03	0.05	3.53	0.00	0.20	0.04	0.00	0.13	0.00
	MAX	31387.92	2.89	0.13	0.86	1607.28	0.46	4.07	1309.29	973.62	0.85	44.53	1.29	143.64	0.04	4.21	0.80	0.03	1.89	0.06
	MIN	18925.88	<LOD	<LOD	0.16	517.01	0.08	1.05	531.61	544.42	0.53	1.80	0.36	73.54	<LOD	<LOD	<LOD	<LOD	<LOD	<LOD
COPD-s	MEDIAN	25616.74	0.66	0.04	0.68	1290.08	0.26	1.11	1224.68	872.30	0.63	7.10	0.84	101.16	0.01	1.63	0.06	0.02	0.36	0.03
	SEM	621.44	0.17	0.01	0.04	80.94	0.04	0.02	54.53	36.06	0.01	1.09	0.08	3.59	0.00	0.20	0.00	0.00	0.07	0.00
	MAX	29707.92	2.83	0.18	1.00	2159.52	0.68	1.28	1722.26	1186.07	0.73	20.36	1.74	142.33	0.03	3.69	0.08	0.02	1.09	0.05
	MIN	17624.57	<LOD	<LOD	0.17	375.66	0.05	0.88	713.06	465.13	0.56	0.76	0.08	70.57	<LOD	<LOD	0.02	0.01	<LOD	0.02
COPD-vs	MEDIAN	21313.86	2.85	0.08	1.33	893.54	0.12	1.35	1053.77	629.07	0.65	14.65	0.62	87.20	0.03	2.32	0.10	0.03	1.28	0.08
	SEM	877.65	0.12	0.01	0.18	87.98	0.01	0.08	55.19	37.49	0.02	0.28	0.12	3.98	0.00	0.13	0.02	0.00	0.19	0.02
	MAX	31005.20	4.00	0.32	5.36	2595.21	0.25	3.49	1763.68	1438.55	1.20	19.16	3.73	153.16	0.07	4.44	0.56	0.08	6.38	0.52
	MIN	10693.36	<LOD	<LOD	0.17	274.44	0.04	0.89	379.53	278.66	0.50	11.78	0.09	37.27	<LOD	1.31	0.03	0.02	0.12	0.02
LOD		0.08	0.06	0.02	0.04	0.05	0.05	0.04	0.05	0.07	0.04	0.03	0.04	0.03	0.01	0.04	0.04	0.02	0.04	0.02

Table S2. Fold changes, p-values from Krustal-Wallis test for significant differences and AUC values from ROC curves calculated using the concentration of elements in human serum. There were not any significant differences found between the comparisons: COPD-mo vs COPD-mi, COPD-se vs COPD-mi, COPD-vs vs COPD-mi, COPD-s vs COPD-mo. Significant differences ($p<0.05$) are shown in the table in red color. FC: fold change; AUC: area under the curve of the receiver operating curve (ROC); NS: not significant; HC: Healthy control; LC: lung cancer; COPD: chronic obstructively pulmonary disease; COPD-LC: patients who developed LC during the follow-up; COPD-vs: very severe COPD; COPD-s: severe COPD, COPD-mo: moderate COPD and COPD-mi: mild COPD.

**Comparison**	**Values**	**Elements**																		
		**Al**	**As**	**Cd**	**Co**	**Cr**	**Cu**	**Fe**	**Mg**	**Mn**	**Mo**	**Ni**	**Pb**	**Sb**	**Se**	**Tl**	**U**	**V**	**W**	**Zn**
LC/HC	FC	2.35	1.35	1.23	1.16	1.82	0.74	0.99	0.96	1.26	0.71	1.16	1.87	0.80	0.96	0.87	0.74	0.84	1.18	0.80
	p-value	0.00	0.00	NS	NS	NS	NS	NS	NS	0.00	NS	NS	0.00	NS	NS	NS	NS	NS	NS	NS
	AUC	0.82	0.77	-	-	-	-	-	-	0.70	-	-	0.89	-	-	-	-	-	-	-
COPD/HC	FC	0.81	0.68	0.59	1.20	0.88	1.00	0.89	1.38	0.81	0.94	1.09	0.92	1.33	1.05	0.67	0.56	0.91	1.31	1.04
	p	NS	0.00	0.02	NS	NS	NS	NS	0.00	NS	NS	NS	NS	0.01	NS	0.04	NS	NS	NS	NS
	AUC	-	<0.70	<0.70	-	-	-	-	0.73	-	-	-	-	<0.70	-	<0.70	-	-	-	-
LC/COPD	FC	4.03	2.16	2.51	0.99	2.65	0.69	1.12	0.67	1.60	0.86	1.25	2.74	0.57	0.89	1.25	1.29	1.04	1.16	0.75
	p-value	0.00	0.00	0.00	NS	0.01	0.00	NS	0.00	0.00	NS	NS	0.00	0.00	NS	0.01	NS	NS	NS	0.00
	AUC	0.88	0.96	<0.70	-	0.91	0.78	-	0.84	0.83	-	-	0.90	0.79	-	-	-	-	-	<0.70
COPD-LC/HC	FC	0.02	0.23	0.08	1.12	0.63	1.04	1.00	1.60	0.41	0.82	1.37	0.04	1.37	1.33	0.57	0.34	0.42	0.72	1.54
	p-value	0.00	0.00	0.01	NS	NS	NS	NS	0.00	0.00	NS	0.00	0.00	NS	0.00	NS	NS	NS	NS	NS
	AUC	0.71	0.79	0.83	-	0.74	-	-	0.80	0.89	-	0.89	0.90	-	0.78	-	-	-	-	-
COPD-mi/HC	FC	0.71	0.63	0.63	1.84	0.73	1.01	0.91	1.41	0.88	0.84	0.69	0.82	1.39	1.04	0.76	0.42	1.02	0.76	1.06
	p-value	NS	0.00	NS	NS	NS	NS	NS	0.00	NS	NS	NS	NS	NS	NS	NS	NS	NS	NS	NS
	AUC	-	0.71	-	-	-	-	-	0.71	-	-	-	-	-	-	-	-	-	-	-
COPD-mo/HC	FC	0.47	0.76	0.41	1.25	0.64	1.17	0.85	1.67	0.43	0.88	0.80	0.75	1.71	1.28	0.61	0.37	0.81	1.96	1.07
	p-value	NS	NS	NS	NS	NS	NS	NS	0.00	0.00	NS	NS	NS	NS	NS	NS	NS	NS	NS	NS
	AUC	-	-	-	-	0.72	0.72	-	0.84	0.91	-	-	-	-	-	-	-	-	-	-
COPD-s/HC	FC	0.22	0.43	0.20	1.65	0.63	1.32	1.34	1.65	0.66	1.28	0.51	0.31	1.20	1.24	0.50	0.39	0.69	0.83	1.32
	p-value	NS	NS	NS	NS	NS	NS	NS	0.00	NS	NS	NS	NS	NS	NS	NS	NS	NS	NS	NS
	AUC	-	-	-	-	-	-	-	0.85	-	-	-	-	-	-	-	-	-	-	-
COPD-vs/ HC	FC	0.94	0.89	0.80	0.74	0.65	1.14	0.93	1.37	1.29	0.95	0.62	1.12	1.71	1.07	0.84	1.14	1.39	1.38	0.95
	p-value	NS	NS	NS	NS	NS	NS	NS	NS	NS	NS	NS	NS	0.01	NS	NS	0.00	NS	0.00	NS
	AUC	-	-	-	-	-	-	-	-	-	-	-	-	0.71	-	-	0.73	-	0.73	-
LC/COPD-LC	FC	109.90	5.94	15.11	1.21	2.88	0.73	1.09	0.62	3.32	0.96	0.70	43.97	0.58	0.72	1.52	2.24	2.40	1.65	0.52
	p-value	0.00	0.00	0.00	NS	NS	0.01	NS	0.00	0.00	NS	NS	0.00	0.00	0.02	0.01	NS	NS	NS	0.00
	AUC	0.94	1.00	0.85	-	-	0.75	-	0.93	0.99	-	-	0.99	0.80	0.80	0.83	0.77		0.71	0.87
LC/COPD-mi	FC	3.33	2.15	1.96	0.63	2.48	0.73	1.09	0.68	1.43	0.84	1.66	2.28	0.57	0.92	1.14	1.76	0.82	1.57	0.75
	p-value	0.00	0.00	0.01	NS	NS	0.00	NS	0.00	0.00	NS	NS	0.00	0.00	NS	NS	NS	NS	NS	NS
	AUC	0.87	0.97	-	-	0.85	0.73	-	-	0.78	-	-	0.86	0.74	-	-	-	-	-	-
LC/COPD-mo	FC	4.97	1.78	2.98	0.92	2.87	0.63	1.16	0.57	2.92	0.80	1.44	2.50	0.47	0.75	1.41	2.01	1.03	0.60	0.75
	p-value	0.00	0.00	NS	NS	NS	0.00	NS	0.00	0.00	NS	NS	0.00	0.00	NS	NS	NS	NS	NS	NS
	AUC	0.89	0.88	0.70	-	-	0.82		0.96	0.99			0.94	0.83	0.78	0.75	-	-		0.76

Table S2. *Cont.*

Comparison	Values	Elements																		
		**Al**	**As**	**Cd**	**Co**	**Cr**	**Cu**	**Fe**	**Mg**	**Mn**	**Mo**	**Ni**	**Pb**	**Sb**	**Se**	**Tl**	**U**	**V**	**W**	**Zn**
LC/COPD-s	FC	10.77	3.15	6.20	0.70	2.87	0.56	0.74	0.58	1.89	0.55	2.26	6.00	0.66	0.77	1.72	1.89	1.21	1.42	0.60
	p-value	0.00	0.00	0.01	NS	NS	0.00	NS	0.00	0.00	NS	NS	0.00	NS	NS	0.00	NS	NS	NS	0.00
	AUC	0.93	0.95	0.80	-	-	0.89	-	-	0.95	-	-	0.95	-	-	0.94	-	-		0.85
LC/COPD-vs	FC	2.50	1.53	1.54	1.57	2.79	0.65	1.07	0.70	0.97	0.75	1.86	1.67	0.47	0.90	1.03	0.65	0.60	0.86	0.84
	p-value	0.00	0.00	NS	NS	NS	0.00	NS	0.00	NS	NS	NS	0.00	0.00	NS	NS	NS	NS	NS	NS
	AUC	0.83	0.97	-	-	0.92	0.80		0.78	0.70			0.86	0.91	-	-	-	-	-	-
COPD-LC/COPD-mi	FC	0.03	0.36	0.14	0.61	0.85	1.02	1.10	1.13	0.46	0.98	1.97	0.06	0.99	1.28	0.75	0.80	0.41	0.80	1.45
	p-value	0.00	0.00	NS	NS	NS	NS	NS	NS	NS	NS	0.00	0.01	NS	0.01	NS	NS	NS	NS	0.01
	AUC	0.84	0.79	0.76	-	-	-	-	-	0.77	-	-	-	-	0.80	-	-	-	-	0.72
COPD-LC/COPD-mo	FC	0.05	0.30	0.19	0.90	0.98	0.88	1.17	0.96	0.95	0.93	1.70	0.06	0.80	1.04	0.93	0.91	0.51	0.36	1.45
	p	0.00	NS	NS	NS	NS	NS	NS	NS	NS	NS	0.00	0.01	NS	NS	NS	NS	NS	NS	NS
	AUC	0.84	0.79	0.77	0.74	-	-	-	-	-	-	0.88	0.79	-	-	-	-	-	-	0.77
COPD-LC/COPD-s	FC	0.10	0.53	0.40	0.68	0.99	0.78	0.75	0.97	0.62	0.64	2.68	0.14	1.14	1.07	1.13	0.86	0.60	0.86	1.17
	p	NS	NS	NS	NS	NS	NS	NS	NS	NS	NS	0.00	NS	NS	NS	NS	NS	NS	NS	NS
	AUC	-	-	-	-	-	0.74	-	-	0.70	-	1.00	0.79	-	-	-	-	-	-	-
COPD-LC/COPD-vs	FC	0.02	0.26	0.11	1.52	0.96	0.91	1.08	1.17	0.32	0.87	2.20	0.05	0.80	1.24	0.68	0.29	0.31	0.52	1.62
	p	0.00	0.00	0.00	NS	NS	NS	NS	NS	0.02	NS	0.00	0.00	NS	0.02	NS	NS	NS	NS	0.00
	AUC	0.98	1.00	0.91	-	-	-	-	-	0.85	-	0.96	0.99	-	0.80	-	-	-	-	0.81
COPD-vs/COPD-mo	FC	1.99	1.16	1.94	0.59	1.03	0.97	1.09	0.82	3.00	1.07	0.77	1.49	1.00	0.84	1.37	3.11	1.71	0.70	0.89
	p	0.00	NS	NS	NS	NS	NS	NS	NS	NS	NS	NS	NS	NS	NS	NS	NS	NS	NS	NS
	AUC	0.79	-	-	-	-	-	-	-	-	-	-	-	-	-	-	-	-	-	-
COPD-vs/COPD-s	FC	4.31	2.06	4.03	0.45	1.03	0.86	0.69	0.83	1.95	0.74	1.22	3.58	1.42	0.86	1.67	2.92	2.01	1.65	0.72
	p	0.00	0.00	0.00	NS	NS	NS	NS	NS	NS	NS	NS	NS	NS	NS	NS	NS	NS	NS	NS
	AUC	0.91	0.79	0.85	-	-	-	-	-	-	-	-	-	-	-	-	-	-	-	-
COPD-mo/COPD-mi	FC	0.67	1.21	0.66	0.68	0.86	1.16	0.94	1.18	0.49	1.05	1.15	0.91	1.23	1.23	0.81	0.87	0.80	2.60	1.00
	p	NS	NS	NS	NS	NS	NS	NS	NS	NS	NS	NS	NS	NS	NS	NS	NS	NS	NS	NS
	AUC	-	-	-	-	-	-	-	-	-	-	-	-	-	-	-	-	-	-	-
COPD-s/COPD-mi	FC	0.31	0.68	0.32	0.89	0.86	1.31	1.47	1.17	0.75	1.52	0.73	0.38	0.87	1.19	0.66	0.93	0.68	1.11	1.25
	p	NS	NS	NS	NS	NS	NS	NS	NS	NS	NS	NS	NS	NS	NS	NS	NS	NS	NS	NS
	AUC	-	-	-	-	-	-	-	-	-	-	-	-	-	-	-	-	-	-	-
COPD-vs/COPD-mi	FC	1.33	1.41	1.27	0.40	0.89	1.12	1.02	0.97	1.47	1.13	0.89	1.36	1.23	1.03	1.11	2.71	1.37	1.83	0.90
	p	NS	NS	NS	NS	NS	NS	NS	NS	NS	NS	NS	NS	NS	NS	NS	NS	NS	NS	NS
	AUC	-	-	-	-	-	-	-	-	-	-	-	-	-	-	-	-	-	-	-
COPD-s/COPD-mo	FC	0.46	0.56	0.48	1.31	1.00	1.13	1.57	0.99	1.54	1.45	0.64	0.42	0.71	0.97	0.82	1.06	0.85	0.43	1.24
	p	NS	NS	NS	NS	NS	NS	NS	NS	NS	NS	NS	NS	NS	NS	NS	NS	NS	NS	NS
	AUC	-	-	-	-	-	-	-	-	-	-	-	-	-	-	-	-	-	-	-

Table S3. VIP values of the different partial least square discriminant analyses. HC: Healthy control; LC: lung cancer; COPD: chronic obstructively pulmonary disease; COPD-LC: patients who developed LC during the follow-up; COPD-vs: very severe COPD; COPD-s: severe COPD, COPD-mo: moderate COPD and COPD-mi: mild COPD. **Bold numbers** indicate significant VIP values greater than 1.

Elements	LC and HC	COPD and LC	COPD-LC and COPD	COPD-mi and HC	COPD-s and HC	COPD-mo and HC	COPD-vs and HC	COPD-mi and COPD-vs
**Al**	**2.06**	**1.89**	**1.58**	0.69	**1.48**	**1.26**	0.31	**1.21**
**As**	**1.23**	**1.60**	**1.28**	**1.24**	**1.13**	0.94	0.26	**1.44**
**Cd**	0.87	0.91	0.93	**2.04**	**1.12**	**1.04**	0.83	**1.37**
**Co**	0.58	0.16	0.67	0.70	0.65	0.29	**1.42**	**1.13**
**Cr**	0.82	0.67	0.39	0.56	0.51	0.53	**1.11**	0.51
**Cu**	0.90	**1.16**	0.30	0.66	**1.26**	**1.05**	**1.51**	0.67
**Fe**	0.55	0.49	0.24	0.49	0.86	0.95	0.29	0.45
**Mg**	0.62	**1.29**	0.39	**1.16**	**1.25**	**1.43**	0.76	0.83
**Mn**	**1.06**	0.78	0.72	**1.27**	0.93	**1.68**	**1.04**	0.87
**Mo**	0.51	0.47	0.20	0.73	0.35	0.48	0.50	0.33
**Ni**	0.80	0.86	**2.64**	0.80	**1.05**	0.39	0.95	0.56
**Pb**	**2.06**	**1.60**	**1.17**	0.58	**1.14**	**1.24**	0.61	**1.01**
**Sb**	0.96	**1.31**	0.90	0.63	0.58	**1.19**	**1.54**	0.85
**Se**	0.87	0.81	0.72	**1.03**	0.70	0.78	0.94	0.57
**Tl**	0.28	0.60	0.68	0.98	**1.21**	0.73	**1.18**	0.40
**U**	0.27	0.41	0.56	**1.22**	**1.07**	**1.12**	**1.50**	**2.00**
**V**	0.71	0.39	0.92	0.64	0.50	0.79	0.30	0.52
**W**	0.79	0.25	0.39	**1.44**	**1.20**	0.95	**1.09**	**1.23**
**Zn**	0.87	**1.04**	**1.04**	0.70	**1.09**	0.96	**1.09**	**1.22**

Table S4. Elemental ratios fold changes of different groups versus HC. Significant p-values are shown in red color. NS: Non-significant. HC: Healthy control; LC: lung cancer; COPD: chronic obstructively pulmonary disease; COPD-LC: patients who developed LC during the follow-up; COPD-vs: very severe COPD; COPD-s: severe COPD, COPD-mo: moderate COPD and COPD-mi: mild COPD.

Ratios	LC/HC		COPD/HC		LC/COPD		COPD-LC /HC		COPD-mo/HC		COPD-se/HC		COPD-vs/HC	
	FC	p	FC	p	FC	p	FC	p	FC	p	FC	p	FC	p
**Al/As**	2.27	0.00	1.05	NS	2.16	0.00	0.25	NS	0.72	NS	0.44	NS	1.30	NS
**Al/Co**	4.28	0.00	0.97	NS	4.41	0.00	0.04	0.00	0.58	NS	0.65	NS	1.78	NS
**Al/Cr**	2.53	0.00	0.91	NS	2.77	0.00	0.04	0.00	0.77	NS	0.34	NS	1.47	NS
**Al/Cu**	4.20	0.00	0.66	NS	6.36	0.00	0.02	0.00	0.45	NS	0.21	NS	1.05	NS
**Al/Fe**	2.50	0.00	0.73	NS	3.43	0.00	0.02	NS	0.49	NS	0.15	NS	1.31	NS
**Al/Mg**	3.96	0.00	0.66	NS	6.03	0.00	0.02	0.01	0.43	NS	0.23	NS	1.10	NS
**Al/Mn**	2.54	0.00	1.15	NS	2.21	0.00	0.10	0.00	1.84	NS	0.43	NS	1.56	NS
**Al/Mo**	3.79	0.00	0.85	NS	4.44	0.00	0.05	0.01	0.62	NS	0.23	NS	1.55	NS
**Al/Ni**	2.78	0.01	1.17	NS	2.37	0.00	0.02	0.00	0.79	NS	0.46	NS	1.96	0.00
**Al/Pb**	1.77	0.00	1.16	NS	1.53	0.01	0.00	0.00	0.50	NS	1.34	NS	1.68	0.00
**Al/Sb**	5.60	0.00	0.71	NS	7.91	0.00	0.03	0.01	0.41	NS	0.17	NS	1.11	NS
**Al/Se**	4.15	0.00	0.87	NS	4.77	0.00	0.02	0.00	0.61	NS	0.35	NS	1.41	NS
**Al/Tl**	2.26	0.00	0.69	NS	3.26	0.00	0.04	0.00	0.49	NS	0.38	NS	0.96	NS
**Al/U**	2.07	0.00	0.61	NS	3.40	0.00	0.02	0.00	0.88	NS	0.28	NS	0.56	NS
**Al/V**	4454.28	NS	1.46	NS	3051.94	NS	0.02	NS	0.87	NS	0.24	NS	1.42	NS
**Al/W**	1.68	NS	0.68	NS	2.47	0.00	0.02	0.00	0.89	NS	0.43	NS	0.68	NS
**Al/Zn**	4.31	0.00	0.83	NS	5.22	0.00	0.02	0.00	0.65	NS	0.28	NS	1.40	NS
**As/Co**	1.91	NS	0.88	NS	2.17	0.00	0.40	NS	0.77	NS	0.89	NS	1.42	NS
**As/Cr**	0.97	NS	0.65	NS	1.50	0.00	0.25	NS	0.75	NS	0.44	NS	0.94	NS
**As/Cu**	2.11	0.00	0.60	0.00	3.50	0.00	0.23	0.00	0.59	NS	0.34	NS	0.87	NS
**As/Fe**	1.60	NS	0.72	NS	2.20	0.00	0.21	0.01	0.69	NS	0.32	NS	1.19	NS
**As/Mg**	0.65	NS	0.17	NS	3.78	0.00	0.05	NS	0.18	NS	0.10	NS	0.26	NS
**As/Mn**	1.05	NS	0.99	NS	1.06	NS	0.57	NS	2.04	NS	0.72	NS	1.17	NS
**As/Mo**	1.85	NS	1.09	NS	1.70	0.01	0.59	NS	1.05	NS	1.20	NS	1.72	NS
**As/Ni**	1.89	NS	1.19	NS	1.59	NS	0.20	0.00	1.29	NS	0.95	NS	1.81	0.00
**As/Sb**	2.67	0.00	0.69	NS	3.85	0.00	0.30	NS	0.56	NS	0.37	NS	1.00	NS
**As/Se**	1.59	0.00	0.66	NS	2.42	0.00	0.19	0.00	0.62	NS	0.44	NS	0.99	NS
**As/Tl**	1.02	NS	0.59	NS	1.73	0.00	0.28	0.00	0.67	NS	0.52	NS	0.74	NS
**As/U**	1.23	NS	0.72	NS	1.70	0.00	0.30	NS	1.37	NS	0.56	NS	0.62	NS
**As/V**	299.67	NS	0.13	NS	2247.86	NS	0.02	NS	0.11	NS	0.03	NS	0.13	NS
**As/W**	1.14	NS	0.80	NS	1.43	NS	0.23	0.00	1.55	NS	0.65	NS	0.75	NS
**As/Zn**	1.95	0.00	0.71	NS	2.76	0.00	0.16	0.00	0.83	NS	0.42	NS	1.11	NS
**Cd/Co**	2.05	NS	0.06	NS	0.60	0.00	0.14	0.01	0.38	NS	0.28	NS	1.16	NS
**Cd/Cr**	1.16	NS	0.67	NS	1.73	NS	0.11	0.01	0.53	NS	0.27	NS	1.03	NS
**Cd/Cu**	0.54	NS	0.12	NS	4.63	0.00	0.03	NS	0.08	NS	0.03	NS	0.17	NS
**Cd/Fe**	0.59	NS	0.23	NS	2.52	0.00	0.03	NS	0.17	NS	0.06	NS	0.42	NS
**Cd/Mg**	0.23	NS	0.04	NS	5.16	0.00	0.01	NS	0.03	NS	0.01	NS	0.07	NS

Table S4. *Cont.*

Ratios	LC/HC		COPD/HC		LC/COPD		COPD-LC /HC		COPD-mo/HC		COPD-se/HC		COPD-vs/HC	
	FC	p	FC	p	FC	p	FC	p	FC	p	FC	p	FC	p
**Cd/Pb**	0.48	NS	0.88	NS	0.54	NS	0.00	NS	0.16	NS	0.42	NS	0.60	NS
**Cd/Sb**	1.98	NS	0.42	NS	4.69	0.00	0.09	NS	0.21	NS	0.10	NS	0.63	NS
**Cd/Mo**	1.56	NS	0.74	NS	2.12	0.00	0.31	NS	0.55	NS	0.18	NS	1.35	NS
**Cd/Ni**	1.25	NS	0.83	NS	1.51	NS	0.07	NS	0.61	NS	0.37	NS	1.39	NS
**Cd/Se**	1.42	NS	0.58	NS	2.44	0.00	0.07	0.01	0.41	NS	0.21	NS	0.91	NS
**Cd/Tl**	1.25	NS	0.57	NS	2.19	0.00	0.13	0.00	0.45	NS	0.37	NS	0.76	NS
**Cd/U**	1.04	NS	0.41	NS	2.51	0.00	0.09	NS	0.65	NS	0.19	NS	0.37	NS
**Cd/V**	4126.23	NS	0.81	NS	5081.50	NS	0.01	NS	0.56	NS	0.12	NS	0.77	NS
**Cd/W**	1.45	NS	0.50	0.00	2.88	0.01	0.08	0.01	0.83	NS	0.28	NS	0.55	NS
**Cd/Zn**	1.40	NS	0.38	NS	3.65	0.00	0.05	NS	0.32	NS	0.10	NS	0.63	NS
**Co/Cr**	0.62	NS	0.85	NS	0.72	NS	0.86	NS	0.96	NS	1.26	NS	0.57	NS
**Co/Cu**	0.33	NS	0.27	NS	1.21	NS	0.20	NS	0.25	NS	0.30	NS	0.13	NS
**Co/Mn**	0.69	NS	2.02	NS	0.34	NS	2.54	0.00	3.19	NS	1.89	NS	0.73	NS
**Co/Pb**	1.41	NS	0.15	NS	9.25	NS	0.00	NS	0.05	NS	0.58	NS	0.05	NS
**Co/Sb**	1.30	NS	1.00	NS	1.29	NS	1.05	NS	0.64	NS	0.32	NS	0.66	NS
**Co/Tl**	1.18	NS	1.22	NS	0.97	NS	1.48	NS	1.34	NS	1.77	NS	0.53	NS
**Co/U**	0.75	NS	1.43	NS	0.52	NS	1.22	NS	1.65	NS	1.67	NS	0.41	NS
**Co/W**	0.79	NS	1.31	NS	0.60	NS	0.95	NS	1.39	NS	1.12	NS	0.49	NS
**Cr/Mn**	1.31	NS	1.42	NS	0.92	NS	2.64	0.00	2.01	NS	1.32	NS	0.89	NS
**Cr/Ni**	1.24	NS	1.12	NS	1.11	NS	0.51	NS	1.03	NS	1.38	NS	1.20	NS
**Cr/Sb**	2.44	0.00	0.80	NS	3.03	0.00	1.03	NS	0.56	NS	0.44	NS	0.72	NS
**Cr/U**	1.48	NS	0.94	NS	1.58	NS	1.00	NS	1.16	NS	0.93	NS	0.57	NS
**Cu/Fe**	0.79	NS	1.19	NS	0.67	0.00	1.08	NS	1.34	NS	1.14	NS	1.38	NS
**Cu/Mg**	0.17	NS	0.18	NS	0.94	NS	0.14	NS	0.15	NS	0.17	NS	0.18	NS
**Cu/Mn**	0.47	0.00	1.77	NS	0.26	0.00	3.20	0.00	2.71	0.00	2.29	NS	1.28	NS
**Cu/Mo**	1.00	NS	1.92	NS	0.52	NS	3.36	NS	1.45	NS	2.38	NS	2.03	0.00
**Cu/Ni**	0.82	NS	1.59	0.00	0.52	0.01	0.69	NS	1.52	NS	2.33	0.00	1.83	0.00
**Cu/Pb**	0.16	NS	0.15	NS	1.04	NS	0.00	NS	0.06	NS	0.70	NS	0.06	NS
**Cu/Se**	0.77	NS	0.90	NS	0.85	NS	0.76	NS	0.87	NS	1.03	NS	1.00	NS
**Cu/Tl**	0.49	0.00	0.94	NS	0.52	0.00	0.99	NS	1.12	NS	1.42	NS	0.81	NS
**Cu/U**	0.72	NS	1.34	NS	0.54	0.00	1.48	NS	1.90	NS	1.66	NS	0.86	NS
**Cu/W**	0.71	NS	1.29	NS	0.55	0.00	1.09	NS	1.72	NS	1.49	NS	1.00	NS
**Cu/Zn**	0.95	NS	1.03	NS	0.92	NS	0.76	NS	1.13	NS	1.08	NS	1.24	NS
**Fe/Mn**	0.70	NS	1.55	NS	0.45	0.00	2.91	0.00	2.11	NS	1.69	NS	1.13	NS
**Fe/Ni**	1.24	NS	1.32	NS	0.94	NS	0.65	NS	1.05	NS	2.28	0.00	1.42	NS
**Fe/Sb**	1.80	0.00	0.95	NS	1.90	0.00	1.34	NS	0.62	NS	0.68	NS	0.87	NS
**Fe/U**	1.13	NS	1.32	NS	0.85	NS	1.49	NS	1.61	NS	1.86	NS	0.85	NS
**Fe/Zn**	1.48	0.01	0.93	NS	1.59	0.00	0.73	NS	0.87	NS	1.11	NS	1.06	NS
**Mg/Mn**	0.61	0.00	2.38	0.00	0.25	0.00	4.90	0.00	3.84	0.00	2.53	0.00	1.52	NS

Table S4. *Cont.*

Ratios	LC/HC		COPD/HC		LC/COPD		COPD-LC /HC		COPD-mo/HC		COPD-se/HC		COPD-vs/HC	
	FC	p	FC	p	FC	p	FC	p	FC	p	FC	p	FC	p
**Mg/Mo**	1.06	NS	1.86	NS	0.57	NS	3.31	0.01	1.50	NS	2.16	NS	1.81	NS
**Mg/Ni**	0.85	NS	1.55	0.00	0.55	0.01	0.79	NS	1.58	NS	2.17	0.00	1.62	0.00
**Mg/Pb**	0.73	NS	1.39	NS	0.53	NS	0.05	NS	0.70	NS	5.37	NS	0.50	NS
**Mg/Tl**	0.70	0.01	1.45	0.00	0.48	0.00	1.82	0.00	1.84	0.00	1.98	0.00	1.10	NS
**Mg/U**	0.72	NS	1.39	NS	0.52	0.00	1.71	0.00	2.01	0.00	1.57	NS	0.81	NS
**Mg/W**	0.64	NS	1.28	NS	0.50	0.00	1.25	NS	1.71	NS	1.36	NS	0.91	NS
**Mg/Zn**	1.23	NS	1.31	0.00	0.94	NS	1.11	NS	1.55	NS	1.27	NS	1.44	0.00
**Mn/Ni**	1.53	NS	1.25	NS	1.22	NS	0.27	0.00	0.59	NS	1.21	NS	1.85	0.00
**Mn/Pb**	0.75	NS	0.18	NS	4.17	NS	0.01	NS	0.04	NS	0.44	NS	0.12	NS
**Mn/Sb**	2.14	0.00	0.81	NS	2.64	0.00	0.46	NS	0.35	NS	0.35	NS	1.10	NS
**Mn/Se**	1.46	0.00	0.86	NS	1.69	0.00	0.31	0.00	0.35	0.00	0.57	NS	1.54	NS
**Mn/Tl**	1.08	NS	0.84	NS	1.28	NS	0.54	NS	0.47	NS	0.82	NS	1.15	NS
**Mn/U**	1.06	NS	0.81	NS	1.31	NS	0.50	NS	0.61	NS	0.76	NS	0.88	NS
**Mn/Zn**	0.50	NS	0.28	NS	1.81	0.00	0.07	0.00	0.12	NS	0.15	NS	0.53	NS
**Mo/Ni**	1.00	NS	1.29	NS	0.78	NS	0.49	NS	0.99	NS	2.20	NS	1.50	NS
**Mo/Pb**	0.25	NS	3.35	NS	0.08	NS	0.00	NS	1.79	NS	19.80	NS	0.75	NS
**Mo/Tl**	0.56	NS	0.92	NS	0.61	0.01	0.99	NS	1.12	NS	1.63	NS	0.68	NS
**Mo/U**	0.61	NS	0.84	NS	0.73	NS	0.86	NS	1.04	NS	1.21	NS	0.56	NS
**Ni/Mo**	1.98	NS	1.59	NS	1.24	NS	4.15	0.00	1.05	NS	1.03	NS	1.43	NS
**Ni/Sb**	2.81	0.00	1.09	NS	2.57	0.00	2.54	0.00	1.01	NS	0.42	NS	0.80	NS
**Ni/Se**	1.73	0.01	0.96	NS	1.80	0.00	1.49	NS	0.92	NS	0.63	NS	0.97	NS
**Ni/Tl**	1.15	NS	1.19	NS	0.96	NS	2.51	0.00	1.53	NS	0.98	NS	0.84	NS
**Ni/U**	1.31	NS	1.18	NS	1.11	NS	2.41	0.00	1.50	NS	0.82	NS	0.61	NS
**Ni/W**	0.65	NS	1.04	NS	0.63	0.00	1.67	NS	1.26	NS	0.70	NS	0.66	NS
**Ni/Zn**	0.55	NS	0.72	NS	0.76	NS	0.34	NS	0.26	NS	0.15	NS	0.28	NS
**Pb/Ni**	3.11	NS	1.39	NS	2.23	NS	0.04	0.00	1.25	NS	0.75	NS	2.43	0.00
**Pb/Sb**	4.14	0.00	0.72	NS	5.78	0.00	0.07	NS	0.60	NS	0.15	NS	1.08	NS
**Pb/Se**	3.03	0.00	1.08	NS	2.81	0.00	0.04	0.00	0.90	NS	0.43	NS	1.95	NS
**Pb/Tl**	1.89	0.00	0.77	NS	2.45	0.00	0.10	0.00	0.75	NS	0.53	NS	1.06	NS
**Pb/U**	1.55	NS	0.68	NS	2.29	0.00	0.06	NS	1.27	NS	0.36	NS	0.59	NS
**Pb/V**	3416.23	NS	1.50	NS	2282.75	NS	0.00	0.00	1.23	NS	0.71	NS	1.60	NS
**Pb/W**	1.62	NS	0.81	NS	2.01	0.01	0.06	0.00	1.45	NS	0.49	NS	0.77	NS
**Pb/Zn**	2.21	0.00	0.68	NS	3.24	0.00	0.03	NS	0.65	NS	0.22	NS	1.28	NS
**Sb/Ni**	0.74	NS	1.52	0.00	0.49	0.01	0.68	NS	1.67	NS	1.52	NS	1.97	0.00
**Sb/Pb**	0.22	NS	2.44	NS	0.09	NS	0.10	NS	1.11	NS	8.90	NS	1.31	NS
**Sb/Se**	0.90	NS	1.36	NS	0.66	0.00	1.04	NS	1.33	NS	1.12	NS	1.79	0.00
**Sb/Tl**	0.55	0.00	1.28	NS	0.43	0.00	1.47	NS	1.56	NS	1.37	NS	1.25	NS
**Sb/U**	0.57	0.01	1.22	NS	0.46	0.00	1.23	NS	1.95	NS	1.06	NS	0.88	NS
**Sb/W**	0.54	NS	1.15	NS	0.47	0.00	0.87	NS	1.84	NS	1.15	NS	0.95	NS

Table S4. *Cont.*

Ratios	LC/HC		COPD/HC		LC/COPD		COPD-LC /HC		COPD-mo/HC		COPD-se/HC		COPD-vs/HC	
	FC	p	FC	p	FC	p	FC	p	FC	p	FC	p	FC	p
**Sb/Zn**	1.02	NS	1.37	NS	0.74	NS	0.82	NS	1.58	NS	1.04	NS	1.97	0.00
**Se/Tl**	0.73	0.00	1.11	NS	0.66	0.00	1.57	0.00	1.42	NS	1.48	NS	0.85	NS
**Se/U**	0.78	NS	1.13	NS	0.69	0.00	1.53	NS	1.65	NS	1.26	NS	0.66	NS
**Se/W**	0.89	NS	1.06	NS	0.84	NS	1.18	NS	1.50	NS	1.06	NS	0.76	NS
**Tl/U**	1.08	NS	1.12	NS	0.96	NS	1.12	NS	1.56	NS	0.90	NS	0.75	NS
**Tl/Zn**	0.11	NS	0.21	NS	0.54	NS	0.04	NS	0.06	NS	0.04	NS	0.09	NS
**U/V**	156.93	NS	0.22	NS	701.65	NS	0.07	NS	0.31	NS	0.09	NS	0.31	NS
**U/W**	1.32	NS	1.31	NS	1.01	NS	0.92	NS	1.01	NS	1.07	NS	1.56	0.00
**W/Zn**	2.25	0.01	1.74	NS	1.30	NS	0.63	NS	1.80	NS	0.79	NS	1.66	NS

Table S5. Elemental ratios fold changes of different groups versus LC. Significant p-values are shown in red color. NS: Non-significant. HC: Healthy control; LC: lung cancer; COPD: chronic obstructively pulmonary disease; COPD-LC: patients who developed LC during the follow-up; COPD-vs: very severe COPD; COPD-s: severe COPD, COPD-mo: moderate COPD and COPD-mi: mild COPD.

Ratios	LC/ COPD-LC		LC/COPD-mi		LC/COPD-mi		LC/COPD-s		LC/COPD-vs	
	FC	p	FC	p	FC	p	FC	p	FC	p
**Al/As**	9.26	0.00	1.58	NS	3.17	0.00	5.11	0.00	1.75	0.00
**Al/Co**	107.38	0.00	4.39	0.00	7.37	NS	6.60	NS	2.41	NS
**Al/Cr**	71.28	0.00	2.35	0.00	3.30	0.00	7.46	0.00	1.72	0.00
**Al/Cu**	168.24	0.00	5.14	0.00	9.41	0.00	19.99	0.00	3.99	0.00
**Al/Fe**	110.10	0.00	3.01	0.00	5.07	0.01	16.75	0.00	1.90	NS
**Al/Mg**	171.68	0.00	5.12	0.00	9.25	0.01	17.41	0.00	3.59	0.00
**Al/Mn**	24.63	0.00	1.96	0.00	1.38	NS	5.96	0.00	1.63	NS
**Al/Mo**	73.77	0.00	4.07	0.00	6.14	NS	16.59	0.01	2.45	NS
**Al/Ni**	135.07	NS	2.02	NS	3.52	NS	6.03	NS	1.42	NS
**Al/Pb**	0.00	0.00	1.31	NS	3.54	0.00	1.33	NS	1.05	NS
**Al/Sb**	178.78	0.00	6.08	0.00	13.78	0.00	33.60	0.00	5.06	0.00
**Al/Se**	177.10	0.00	4.00	0.00	6.85	0.00	11.72	0.00	2.94	0.00
**Al/Tl**	62.01	0.00	2.53	0.00	4.63	0.00	5.93	0.00	2.34	0.00
**Al/U**	87.00	0.00	2.36	0.00	2.34	0.01	7.29	0.00	3.73	0.00
**Al/W**	84.18	0.00	1.80	NS	1.89	NS	3.93	NS	2.46	0.01
**Al/Zn**	200.75	0.00	4.56	0.00	6.67	0.00	15.36	0.00	3.08	0.00
**As/Co**	4.81	NS	2.60	0.00	2.49	NS	2.15	NS	1.35	NS
**As/Cr**	3.92	0.00	1.51	0.00	1.30	NS	2.22	0.00	1.03	NS
**As/Cu**	9.17	0.00	3.30	0.00	3.54	0.00	6.20	0.00	2.42	0.00
**As/Fe**	7.46	0.00	2.18	0.00	2.31	NS	4.96	0.01	1.34	NS
**As/Mg**	11.90	0.00	3.75	0.00	3.61	NS	6.23	0.01	2.48	0.00
**As/Mn**	1.84	0.00	1.24	NS	0.51	0.01	1.46	NS	0.89	NS
**As/Mo**	3.14	NS	2.19	0.00	1.77	NS	1.55	NS	1.08	NS
**As/Ni**	9.61	NS	1.58	NS	1.47	NS	1.99	NS	1.04	NS
**As/Sb**	8.76	0.00	3.51	0.00	4.77	0.00	7.12	0.00	2.68	0.00
**As/Se**	8.24	0.00	2.33	0.00	2.57	0.00	3.65	0.00	1.60	0.00
**As/Tl**	3.65	0.00	1.67	0.00	1.52	NS	1.97	0.00	1.38	NS
**As/U**	4.17	0.00	1.49	NS	0.90	NS	2.19	NS	1.97	0.00
**As/W**	4.89	NS	1.27	NS	0.74	NS	1.76	NS	1.53	NS
**As/Zn**	12.41	0.00	2.81	0.00	2.36	NS	4.59	0.00	1.75	0.00
**Cd/Co**	14.37	NS	2.76	NS	5.39	NS	7.26	NS	1.76	NS
**Cd/Cr**	10.35	0.01	1.47	NS	2.19	NS	4.23	NS	1.13	NS
**Cd/Cu**	18.75	NS	3.68	NS	6.65	NS	16.49	NS	3.19	NS
**Cd/Fe**	18.36	0.01	2.32	NS	3.54	NS	9.68	NS	1.42	NS
**Cd/Mg**	35.65	NS	4.31	NS	7.42	NS	16.97	NS	3.24	NS
**Cd/Mo**	5.05	NS	2.23	NS	2.82	NS	8.60	NS	1.16	NS
**Cd/Ni**	18.41	NS	1.36	NS	2.06	NS	3.34	NS	0.90	NS

Table S5. *Cont.*

Ratios	LC/ COPD-LC		LC/COPD-mi		LC/COPD-mi		LC/COPD-s		LC/COPD-vs	
	FC	p	FC	p	FC	p	FC	p	FC	p
**Cd/Pb**	0.00	NS	0.28	NS	2.95	NS	1.14	NS	0.80	NS
**Cd/Sb**	22.79	0.01	3.59	0.00	9.45	NS	20.57	NS	3.14	0.01
**Cd/Se**	19.20	0.00	2.04	0.01	3.43	NS	6.62	0.01	1.56	NS
**Cd/Tl**	9.88	0.00	1.80	NS	2.80	NS	3.37	NS	1.64	NS
**Cd/U**	11.77	0.01	1.85	NS	1.59	NS	5.59	NS	2.79	NS
**Cd/W**	17.14	NS	2.38	NS	1.75	NS	5.19	NS	2.64	NS
**Cd/Zn**	30.13	NS	3.16	NS	4.42	NS	13.44	NS	2.23	NS
**Co/Cr**	0.71	NS	0.69	NS	0.64	NS	0.49	0.01	1.08	NS
**Co/Cu**	1.60	NS	0.85	NS	1.30	NS	1.09	NS	2.60	0.00
**Co/Mn**	0.27	0.00	0.29	NS	0.22	0.00	0.37	0.00	0.95	NS
**Co/Pb**	627.89	NS	7.79	NS	31.20	NS	2.44	NS	27.26	NS
**Co/Sb**	1.24	NS	0.88	NS	2.04	0.01	4.05	0.00	1.96	0.00
**Co/Tl**	0.80	NS	0.86	NS	0.88	NS	0.67	NS	2.22	NS
**Co/U**	0.62	NS	0.37	NS	0.45	0.00	0.45	0.01	1.84	0.01
**Co/W**	0.84	NS	0.39	NS	0.57	NS	0.71	NS	1.62	NS
**Cr/Mn**	0.50	NS	1.09	NS	0.65	NS	0.99	NS	1.47	NS
**Cr/Ni**	2.43	NS	1.00	NS	1.21	NS	0.90	NS	1.03	NS
**Cr/Sb**	2.36	NS	2.61	0.00	4.36	NS	5.56	NS	3.38	0.00
**Cr/U**	1.48	NS	1.35	NS	1.27	NS	1.59	NS	2.62	NS
**Cu/Fe**	0.73	NS	0.74	NS	0.59	0.01	0.70	NS	0.58	0.00
**Cu/Mg**	1.23	NS	0.80	NS	1.14	NS	1.02	NS	0.98	NS
**Cu/Mn**	0.15	0.00	0.39	0.00	0.17	0.00	0.20	0.00	0.37	0.00
**Cu/Mo**	0.30	NS	0.76	NS	0.69	NS	0.42	NS	0.49	0.00
**Cu/Ni**	1.19	NS	0.52	NS	0.54	NS	0.35	NS	0.45	NS
**Cu/Pb**	37.86	NS	1.16	NS	2.62	NS	0.23	NS	2.67	NS
**Cu/Se**	1.01	NS	0.88	NS	0.88	NS	0.74	NS	0.77	0.01
**Cu/Tl**	0.49	0.00	0.58	0.00	0.44	0.00	0.35	0.00	0.61	0.00
**Cu/U**	0.49	0.00	0.52	0.00	0.38	0.00	0.43	0.00	0.83	NS
**Cu/W**	0.65	NS	0.51	0.00	0.41	0.01	0.47	NS	0.71	NS
**Cu/Zn**	1.25	NS	1.00	NS	0.84	NS	0.88	NS	0.77	0.00
**Fe/Mn**	0.24	0.00	0.61	NS	0.33	0.00	0.42	0.00	0.62	NS
**Fe/Ni**	1.91	NS	0.95	NS	1.18	NS	0.54	NS	0.87	NS
**Fe/Sb**	1.34	NS	1.78	0.01	2.93	NS	2.64	NS	2.07	0.00
**Fe/U**	0.76	NS	0.84	NS	0.70	NS	0.61	NS	1.34	NS
**Fe/Zn**	2.04	0.01	1.68	0.00	1.70	NS	1.34	NS	1.39	NS
**Mg/Mn**	0.12	0.00	0.38	0.00	0.16	0.00	0.24	0.00	0.40	0.00
**Mg/Mo**	0.32	0.01	0.77	NS	0.71	NS	0.49	NS	0.59	NS
**Mg/Ni**	1.07	NS	0.53	NS	0.54	NS	0.39	NS	0.52	NS
**Mg/Pb**	13.89	NS	0.48	NS	1.04	NS	0.14	NS	1.45	NS

Table S5. *Cont.*

Ratios	LC/ COPD-LC		LC/COPD-mi		LC/COPD-mi		LC/COPD-s		LC/COPD-vs	
	FC	p	FC	p	FC	p	FC	p	FC	p
**Mg/Tl**	0.38	0.00	0.54	0.00	0.38	0.00	0.35	0.00	0.63	0.00
**Mg/U**	0.42	0.00	0.50	0.00	0.36	0.00	0.46	0.00	0.90	NS
**Mg/W**	0.51	0.00	0.46	0.00	0.37	0.00	0.47	0.01	0.71	NS
**Mg/Zn**	1.11	NS	0.98	NS	0.80	NS	0.97	NS	0.85	NS
**Mn/Ni**	5.75	NS	1.09	NS	2.59	NS	1.26	NS	0.83	NS
**Mn/Pb**	82.63	NS	3.04	NS	20.62	NS	1.72	NS	6.37	NS
**Mn/Sb**	4.61	0.00	2.18	0.00	6.05	0.00	6.13	0.00	1.94	0.00
**Mn/Se**	4.78	0.00	1.74	0.00	4.14	0.00	2.57	0.00	0.95	NS
**Mn/Tl**	1.98	0.01	1.27	NS	2.29	NS	1.32	NS	0.94	NS
**Mn/U**	2.12	0.00	1.12	NS	1.75	NS	1.40	NS	1.21	NS
**Mn/Zn**	6.95	0.00	1.91	0.00	4.23	0.00	3.25	0.00	0.95	NS
**Mo/Ni**	2.03	NS	0.80	NS	1.02	NS	0.46	NS	0.67	NS
**Mo/Pb**	0.00	0.00	0.12	NS	0.14	NS	0.01	NS	0.34	0.00
**Mo/Tl**	0.57	NS	0.70	NS	0.50	NS	0.34	0.00	0.83	NS
**Mo/U**	0.72	NS	0.71	NS	0.59	NS	0.51	0.01	1.10	NS
**Ni/Mo**	0.48	NS	1.90	NS	1.89	NS	1.92	NS	1.38	NS
**Ni/Sb**	1.11	NS	2.94	0.00	2.79	NS	6.75	NS	3.51	0.00
**Ni/Se**	1.16	NS	1.99	0.00	1.87	NS	2.76	NS	1.78	NS
**Ni/Tl**	0.46	0.00	1.25	NS	0.75	NS	1.17	NS	1.37	NS
**Ni/U**	0.54	0.00	1.18	NS	0.88	NS	1.60	NS	2.15	NS
**Ni/W**	0.39	0.00	0.60	0.01	0.52	0.01	0.94	NS	0.99	NS
**Ni/Zn**	1.60	NS	0.39	NS	2.15	NS	3.77	NS	1.98	NS
**Pb/Ni**	84.93	NS	2.16	NS	2.49	NS	4.12	NS	1.28	NS
**Pb/Sb**	61.71	0.00	4.57	0.00	6.90	0.00	27.41	0.00	3.84	0.00
**Pb/Se**	70.50	0.00	2.71	0.00	3.37	0.00	7.08	0.00	1.55	NS
**Pb/Tl**	19.53	0.00	2.09	0.00	2.50	0.00	3.58	0.00	1.78	0.00
**Pb/U**	27.44	0.00	1.72	0.00	1.22	NS	4.29	0.00	2.64	0.00
**Pb/V**	0.00	NS	1515.31	NS	2783.77	NS	4842.67	NS	2132.06	NS
**Pb/W**	27.91	NS	1.57	NS	1.12	NS	3.29	NS	2.12	NS
**Pb/Zn**	78.67	0.00	3.26	0.00	3.42	0.00	9.97	0.00	1.73	NS
**Sb/Ni**	1.09	NS	0.49	NS	0.44	NS	0.49	NS	0.38	NS
**Sb/Pb**	2.30	NS	0.09	NS	0.20	0.00	0.03	NS	0.17	0.00
**Sb/Se**	0.87	NS	0.70	NS	0.68	NS	0.81	NS	0.51	0.00
**Sb/Tl**	0.37	0.00	0.48	0.00	0.35	0.00	0.40	0.00	0.44	0.00
**Sb/U**	0.46	0.00	0.43	0.00	0.29	0.00	0.53	NS	0.65	NS
**Sb/W**	0.62	NS	0.44	0.00	0.29	0.00	0.47	NS	0.57	NS
**Sb/Zn**	1.24	NS	0.82	NS	0.65	NS	0.98	NS	0.52	0.00
**Se/Tl**	0.47	0.00	0.78	NS	0.52	0.00	0.49	0.00	0.86	NS
**Se/U**	0.51	0.00	0.69	NS	0.48	0.00	0.62	NS	1.19	NS

Table S5. *Cont.*

Ratios	LC/ COPD-LC		LC/COPD-mi		LC/COPD-mi		LC/COPD-s		LC/COPD-vs	
	FC	p	FC	p	FC	p	FC	p	FC	p
**Se/W**	0.75	NS	0.80	NS	0.59	NS	0.84	NS	1.17	NS
**Tl/U**	0.96	NS	0.83	NS	0.69	NS	1.20	NS	1.44	0.01
**Tl/Zn**	2.64	0.00	0.27	NS	1.94	NS	2.89	0.00	1.24	NS
**U/V**	2200.65	NS	650.61	NS	510.12	NS	1790.13	NS	507.15	NS
**U/W**	1.43	NS	0.93	NS	1.31	NS	1.24	NS	0.84	NS
**W/Zn**	3.55	NS	0.93	NS	1.25	NS	2.86	NS	1.36	NS

Table S6. Elemental ratios fold changes of different groups versus COPD-LC. Significant p-values are shown in red color. NS: Non-significant. HC: Healthy control; LC: lung cancer; COPD: chronic obstructively pulmonary disease; COPD-LC: patients who developed LC during the follow-up; COPD-vs: very severe COPD; COPD-s: severe COPD, COPD-mo: moderate COPD and COPD-mi: mild COPD.

Ratios	COPD-LC /COPD-mi		COPD-LC /COPD-mo		COPD-LC /COPD-se		COPD-LC /COPD-vs	
	FC	p	FC	p	FC	p	FC	p
**Al/As**	0.17	NS	0.34	NS	0.55	NS	0.19	0.00
**Al/Co**	0.04	0.00	0.07	NS	0.06	NS	0.02	0.00
**Al/Cr**	0.03	0.00	0.05	NS	0.10	NS	0.02	0.00
**Al/Cu**	0.03	0.00	0.06	NS	0.12	NS	0.02	0.00
**Al/Fe**	0.03	0.00	0.05	NS	0.15	NS	0.02	0.00
**Al/Mg**	0.03	0.00	0.05	NS	0.10	NS	0.02	0.00
**Al/Mn**	0.08	0.00	0.06	NS	0.24	NS	0.07	0.00
**Al/Mo**	0.06	0.00	0.08	NS	0.22	NS	0.03	0.00
**Al/Ni**	0.01	0.00	0.03	NS	0.04	NS	0.01	0.00
**Al/Pb**	0.00	0.00	0.00	NS	0.00	NS	0.00	0.00
**Al/Sb**	0.03	0.00	0.08	NS	0.19	NS	0.03	0.00
**Al/Se**	0.02	0.00	0.04	NS	0.07	NS	0.02	0.00
**Al/Tl**	0.04	0.00	0.07	NS	0.10	NS	0.04	0.00
**Al/U**	0.03	0.00	0.03	NS	0.08	NS	0.04	0.00
**Al/V**	0.01	NS	0.03	NS	0.09	NS	0.02	0.00
**Al/W**	0.02	0.00	0.02	NS	0.05	NS	0.03	0.00
**Al/Zn**	0.02	0.00	0.03	NS	0.08	NS	0.02	0.00
**As/Co**	0.54	NS	0.52	NS	0.45	NS	0.28	0.00
**As/Cr**	0.38	0.00	0.33	NS	0.57	NS	0.26	0.00
**As/Cu**	0.36	0.00	0.39	NS	0.68	NS	0.26	0.00
**As/Fe**	0.29	0.01	0.31	NS	0.66	NS	0.18	0.00
**As/Mg**	0.32	0.00	0.30	NS	0.52	NS	0.21	0.00
**As/Mn**	0.67	NS	0.28	NS	0.79	NS	0.48	NS
**As/Mo**	0.70	NS	0.57	NS	0.49	NS	0.34	NS
**As/Ni**	0.16	0.00	0.15	NS	0.21	NS	0.11	0.00
**As/Sb**	0.40	0.01	0.54	NS	0.81	NS	0.31	0.00
**As/Se**	0.28	0.00	0.31	NS	0.44	NS	0.19	0.00
**As/Tl**	0.46	0.01	0.42	NS	0.54	NS	0.38	0.00
**As/U**	0.36	0.01	0.21	NS	0.52	NS	0.47	0.01
**As/V**	0.11	NS	0.21	NS	0.75	NS	0.19	0.02
**As/W**	0.26	0.00	0.15	NS	0.36	NS	0.31	0.00
**As/Zn**	0.23	0.00	0.19	NS	0.37	NS	0.14	0.00
**Cd/Co**	0.19	NS	0.38	NS	0.50	NS	0.12	0.00
**Cd/Cr**	0.14	NS	0.21	NS	0.41	NS	0.11	0.00
**Cd/Cu**	0.20	NS	0.35	NS	0.88	NS	0.17	0.00
**Cd/Fe**	0.13	NS	0.19	NS	0.53	NS	0.08	0.00
**Cd/Mg**	0.12	NS	0.21	NS	0.48	NS	0.09	0.00

Table S6. *Cont.*

Ratios	COPD-LC /COPD-mi		COPD-LC /COPD-mo		COPD-LC /COPD-se		COPD-LC /COPD-vs	
	FC	p	FC	p	FC	p	FC	p
**Cd/Mo**	0.44	NS	0.56	NS	1.70	NS	0.23	0.01
**Cd/Ni**	0.07	NS	0.11	NS	0.18	NS	0.05	0.00
**Cd/Pb**	0.00	NS	0.00	NS	0.00	NS	0.00	0.00
**Cd/Sb**	0.16	NS	0.41	NS	0.90	NS	0.14	0.00
**Cd/Se**	0.11	NS	0.18	NS	0.34	NS	0.08	0.00
**Cd/Tl**	0.18	NS	0.28	NS	0.34	NS	0.17	0.00
**Cd/U**	0.16	NS	0.14	NS	0.48	NS	0.24	0.00
**Cd/V**	0.01	NS	0.01	NS	0.06	NS	0.01	0.00
**Cd/W**	0.14	0.01	0.10	NS	0.30	NS	0.15	0.00
**Cd/Zn**	0.10	NS	0.15	NS	0.45	NS	0.07	0.00
**Co/Cr**	0.98	NS	0.90	NS	0.68	NS	1.52	NS
**Co/Cu**	0.53	NS	0.81	NS	0.68	NS	1.62	NS
**Co/Mn**	1.06	NS	0.80	NS	1.34	NS	3.47	0.01
**Co/Pb**	0.01	NS	0.05	NS	0.00	NS	0.04	0.00
**Co/Sb**	0.71	NS	1.65	NS	3.27	NS	1.58	NS
**Co/Tl**	1.07	NS	1.11	NS	0.84	NS	2.79	NS
**Co/U**	0.60	NS	0.74	NS	0.73	NS	2.98	NS
**Co/W**	0.47	NS	0.68	NS	0.85	NS	1.94	NS
**Cr/Mn**	2.20	0.01	1.31	NS	2.00	NS	2.97	0.00
**Cr/Ni**	0.41	0.00	0.50	0.00	0.37	0.00	0.42	0.00
**Cr/Sb**	1.11	NS	1.85	NS	2.36	NS	1.43	NS
**Cr/U**	0.91	NS	0.86	NS	1.07	NS	1.77	0.00
**Cu/Fe**	1.01	NS	0.81	NS	0.95	NS	0.79	NS
**Cu/Mg**	0.65	NS	0.92	NS	0.82	NS	0.79	0.01
**Cu/Mn**	2.67	0.00	1.18	NS	1.40	NS	2.51	0.00
**Cu/Mo**	2.57	NS	2.32	NS	1.41	NS	1.66	NS
**Cu/Ni**	0.44	0.00	0.45	0.00	0.30	0.00	0.38	0.00
**Cu/Pb**	0.03	NS	0.07	NS	0.01	NS	0.07	0.01
**Cu/Se**	0.87	NS	0.87	NS	0.73	NS	0.76	0.02
**Cu/Tl**	1.19	NS	0.89	NS	0.70	NS	1.23	NS
**Cu/U**	1.08	NS	0.78	NS	0.89	NS	1.71	0.00
**Cu/W**	0.79	NS	0.63	NS	0.73	NS	1.09	NS
**Cu/Zn**	0.80	NS	0.67	NS	0.70	NS	0.61	0.00
**Fe/Mn**	2.52	0.00	1.38	NS	1.72	NS	2.57	0.00
**Fe/Ni**	0.50	0.00	0.62	NS	0.28	0.00	0.45	0.01
**Fe/Sb**	1.33	NS	2.18	NS	1.97	NS	1.54	NS
**Fe/U**	1.11	NS	0.93	NS	0.80	NS	1.77	0.02
**Fe/Zn**	0.82	NS	0.84	NS	0.66	NS	0.68	NS
**Mg/Mn**	3.05	0.00	1.28	NS	1.94	NS	3.22	0.00

Table S6. *Cont.*

Ratios	COPD-LC /COPD-mi		COPD-LC /COPD-mo		COPD-LC /COPD-se		COPD-LC /COPD-vs	
	FC	p	FC	p	FC	p	FC	p
**Mg/Mo**	2.40	NS	2.21	NS	1.53	NS	1.83	NS
**Mg/Ni**	0.49	0.00	0.50	0.00	0.36	0.00	0.49	0.00
**Mg/Pb**	0.03	NS	0.08	NS	0.01	NS	0.10	0.01
**Mg/Tl**	1.41	NS	0.99	NS	0.92	NS	1.65	0.00
**Mg/U**	1.19	NS	0.85	NS	1.09	NS	2.12	0.00
**Mg/W**	0.89	NS	0.73	NS	0.91	NS	1.38	NS
**Mg/Zn**	0.89	NS	0.72	NS	0.88	NS	0.77	0.01
**Mn/Ni**	0.19	0.00	0.45	NS	0.22	0.00	0.14	0.00
**Mn/Pb**	0.04	NS	0.25	NS	0.02	NS	0.08	0.01
**Mn/Sb**	0.47	NS	1.31	NS	1.33	NS	0.42	0.00
**Mn/Se**	0.36	0.01	0.87	NS	0.54	NS	0.20	NS
**Mn/Tl**	0.64	NS	1.15	NS	0.67	NS	0.47	NS
**Mn/U**	0.53	NS	0.82	NS	0.66	NS	0.57	NS
**Mn/Zn**	0.27	NS	0.61	NS	0.47	NS	0.14	NS
**Mo/Ni**	0.39	0.00	0.50	NS	0.22	0.00	0.33	NS
**Mo/Pb**	0.00	NS	0.00	NS	0.00	NS	0.00	0.00
**Mo/Tl**	1.24	NS	0.88	NS	0.61	NS	1.46	NS
**Mo/U**	0.99	NS	0.83	NS	0.71	NS	1.54	NS
**Ni/Mo**	4.00	0.01	3.96	NS	4.03	NS	2.89	NS
**Ni/Sb**	2.65	0.00	2.51	NS	6.09	0.00	3.17	0.00
**Ni/Se**	1.71	0.00	1.61	NS	2.37	0.00	1.54	NS
**Ni/Tl**	2.73	0.00	1.64	NS	2.55	0.00	2.99	0.00
**Ni/U**	2.17	0.00	1.61	NS	2.94	0.00	3.95	0.00
**Ni/W**	1.52	NS	1.32	NS	2.39	0.00	2.52	0.00
**Ni/Zn**	0.24	NS	1.34	NS	2.35	0.00	1.24	NS
**Pb/Ni**	0.03	0.00	0.03	NS	0.05	NS	0.02	0.00
**Pb/Sb**	0.07	0.01	0.11	NS	0.44	NS	0.06	0.00
**Pb/Se**	0.04	0.00	0.05	NS	0.10	NS	0.02	NS
**Pb/Tl**	0.11	0.00	0.13	NS	0.18	NS	0.09	0.00
**Pb/U**	0.06	0.01	0.04	NS	0.16	NS	0.10	0.00
**Pb/V**	0.00	NS	0.00	NS	0.00	NS	0.00	NS
**Pb/W**	0.06	0.00	0.04	NS	0.12	NS	0.08	0.00
**Pb/Zn**	0.04	0.00	0.04	NS	0.13	NS	0.02	NS
**Sb/Ni**	0.45	0.00	0.41	NS	0.45	NS	0.34	0.00
**Sb/Pb**	0.04	NS	0.09	NS	0.01	NS	0.07	0.00
**Sb/Se**	0.81	NS	0.79	NS	0.93	NS	0.58	NS
**Sb/Tl**	1.30	NS	0.94	NS	1.08	NS	1.18	NS
**Sb/U**	0.94	NS	0.63	NS	1.16	NS	1.41	NS
**Sb/W**	0.71	NS	0.47	NS	0.76	NS	0.91	NS

Table S6. *Cont.*

Ratios	COPD-LC /COPD-mi		COPD-LC /COPD-mo		COPD-LC /COPD-se		COPD-LC /COPD-vs	
	FC	p	FC	p	FC	p	FC	p
**Sb/Zn**	0.66	NS	0.52	NS	0.79	NS	0.42	0.01
**Se/Tl**	1.67	0.00	1.10	NS	1.06	NS	1.85	0.00
**Se/U**	1.35	NS	0.93	NS	1.21	NS	2.32	0.00
**Se/W**	1.07	NS	0.79	NS	1.11	NS	1.55	0.00
**Tl/U**	0.86	NS	0.72	NS	1.25	NS	1.50	0.01
**Tl/Zn**	0.10	NS	0.74	NS	1.09	NS	0.47	0.01
**U/V**	0.30	NS	0.23	NS	0.81	NS	0.23	0.02
**U/W**	0.65	NS	0.91	NS	0.86	NS	0.59	0.00
**W/Zn**	0.26	NS	0.35	NS	0.81	NS	0.38	0.01

Table S7. Elemental ratios fold changes of different COPD groups. Significant p-values are shown in red color. NS: Non-significant. HC: Healthy control; LC: lung cancer; COPD: chronic obstructively pulmonary disease; COPD-LC: patients who developed LC during the follow-up; COPD-vs: very severe COPD; COPD-s: severe COPD, COPD-mo: moderate COPD and COPD-mi: mild COPD.

Ratios	COPD-vs/COPD-mo		COPD-vs/COPD-se		COPD-vs/COPD-mi		COPD-mo/COPD-mi	
	FC	p	FC	p	FC	p	FC	p
**Al/As**	1.81	NS	2.92	0.00	0.90	NS	0.50	NS
**Al/Co**	3.06	NS	2.74	NS	1.82	NS	0.60	NS
**Al/Cr**	1.92	NS	4.33	0.00	1.37	NS	0.71	NS
**Al/Cu**	2.36	NS	5.01	0.00	1.29	NS	0.55	NS
**Al/Fe**	2.66	NS	8.79	0.00	1.58	NS	0.59	NS
**Al/Mg**	2.58	NS	4.85	0.00	1.43	NS	0.55	NS
**Al/Mn**	0.85	NS	3.66	NS	1.21	NS	1.42	NS
**Al/Mo**	2.50	NS	6.77	0.00	1.66	NS	0.66	NS
**Al/Ni**	2.49	0.00	4.26	0.00	1.43	NS	0.57	NS
**Al/Pb**	3.36	0.00	1.26	NS	1.24	NS	0.37	NS
**Al/Sb**	2.72	0.00	6.64	0.00	1.20	NS	0.44	NS
**Al/Se**	2.33	NS	3.99	0.00	1.36	NS	0.58	NS
**Al/Tl**	1.98	NS	2.53	0.01	1.08	NS	0.55	NS
**Al/U**	0.63	NS	1.96	NS	0.63	NS	1.01	NS
**Al/V**	1.63	NS	5.83	NS	0.57	NS	0.35	NS
**Al/W**	0.77	NS	1.60	NS	0.73	NS	0.95	NS
**Al/Zn**	2.17	NS	4.99	0.00	1.48	NS	0.68	NS
**As/Co**	1.85	NS	1.60	NS	1.93	0.00	1.04	NS
**As/Cr**	1.25	NS	2.14	0.00	1.46	NS	1.16	NS
**As/Cu**	1.46	NS	2.56	0.00	1.36	NS	0.93	NS
**As/Fe**	1.72	NS	3.70	0.00	1.63	NS	0.95	NS
**As/Mg**	1.46	NS	2.52	0.00	1.52	NS	1.04	NS
**As/Mn**	0.57	NS	1.63	NS	1.39	NS	2.42	NS
**As/Mo**	1.65	NS	1.44	NS	2.03	NS	1.23	NS
**As/Ni**	1.41	NS	1.91	0.00	1.51	NS	1.07	NS
**As/Sb**	1.78	NS	2.66	0.00	1.31	NS	0.74	NS
**As/Se**	1.60	NS	2.28	0.00	1.45	NS	0.90	NS
**As/Tl**	1.10	NS	1.43	NS	1.21	NS	1.10	NS

Table S7. *Cont.*

Ratios	COPD-vs/COPD-mo		COPD-vs/COPD-se		COPD-vs/COPD-mi		COPD-mo/COPD-mi	
	FC	p	FC	p	FC	p	FC	p
**As/U**	0.45	NS	1.11	NS	0.75	NS	1.66	NS
**As/V**	1.13	NS	3.97	NS	0.59	NS	0.53	NS
**As/W**	0.48	NS	1.15	NS	0.83	NS	1.72	NS
**As/Zn**	1.35	NS	2.62	0.00	1.60	NS	1.19	NS
**Cd/Co**	3.06	NS	4.11	NS	1.56	NS	0.51	NS
**Cd/Cr**	1.94	NS	3.76	0.01	1.30	NS	0.67	NS
**Cd/Cu**	2.09	NS	5.18	0.00	1.16	NS	0.55	NS
**Cd/Fe**	2.50	NS	6.82	0.01	1.63	NS	0.65	NS
**Cd/Mg**	2.29	NS	5.24	0.00	1.33	NS	0.58	NS
**Cd/Mo**	2.43	NS	7.42	NS	1.93	NS	0.79	NS
**Cd/Ni**	2.30	NS	3.73	NS	1.52	NS	0.66	NS
**Cd/Pb**	3.71	NS	1.43	NS	0.36	NS	0.10	NS
**Cd/Sb**	3.01	NS	6.55	0.00	1.14	NS	0.38	NS
**Cd/Se**	2.20	NS	4.24	0.00	1.31	NS	0.59	NS
**Cd/Tl**	1.70	NS	2.05	NS	1.09	NS	0.64	NS
**Cd/U**	0.57	NS	2.00	NS	0.66	NS	1.16	NS
**Cd/V**	1.38	NS	6.22	NS	0.56	NS	0.41	NS
**Cd/W**	0.67	NS	1.97	NS	0.90	NS	1.36	NS
**Cd/Zn**	1.98	NS	6.04	0.00	1.42	NS	0.72	NS
**Co/Cr**	0.59	NS	0.45	NS	0.64	NS	1.09	NS
**Co/Cu**	0.50	NS	0.42	NS	0.33	NS	0.66	NS
**Co/Mn**	0.23	NS	0.39	NS	0.31	NS	1.33	NS
**Co/Pb**	1.14	NS	0.09	NS	0.29	NS	0.25	NS
**Co/Sb**	1.04	NS	2.06	NS	0.45	NS	0.43	NS
**Co/Tl**	0.40	NS	0.30	0.00	0.39	NS	0.97	NS
**Co/U**	0.25	0.00	0.24	0.00	0.20	NS	0.81	NS
**Co/W**	0.35	0.00	0.44	0.01	0.24	NS	0.69	NS
**Cr/Mn**	0.44	NS	0.67	NS	0.74	NS	1.68	NS

Table S7. *Cont.*

Ratios	COPD-vs/COPD-mo		COPD-vs/COPD-se		COPD-vs/COPD-mi		COPD-mo/COPD-mi	
	FC	p	FC	p	FC	p	FC	p
**Cr/Ni**	1.17	NS	0.87	NS	0.97	NS	0.83	NS
**Cr/Sb**	1.29	NS	1.65	NS	0.77	NS	0.60	NS
**Cr/U**	0.49	0.00	0.61	0.01	0.51	NS	1.06	NS
**Cu/Fe**	1.03	NS	1.21	NS	1.28	NS	1.24	NS
**Cu/Mg**	1.17	NS	1.04	NS	0.82	NS	0.70	NS
**Cu/Mn**	0.47	NS	0.56	NS	1.06	NS	2.26	NS
**Cu/Mo**	1.40	NS	0.85	NS	1.55	NS	1.11	NS
**Cu/Ni**	1.20	NS	0.78	NS	1.15	NS	0.96	NS
**Cu/Pb**	0.98	NS	0.08	NS	0.43	NS	0.44	NS
**Cu/Se**	1.14	NS	0.96	NS	1.14	NS	1.00	NS
**Cu/Tl**	0.72	NS	0.57	0.00	0.96	NS	1.33	NS
**Cu/U**	0.45	0.00	0.52	0.00	0.63	NS	1.39	NS
**Cu/W**	0.58	NS	0.67	NS	0.73	NS	1.25	NS
**Cu/Zn**	1.09	NS	1.14	NS	1.30	NS	1.19	NS
**Fe/Mn**	0.54	NS	0.67	NS	0.98	NS	1.82	NS
**Fe/Ni**	1.36	NS	0.62	NS	1.10	NS	0.81	NS
**Fe/Sb**	1.41	NS	1.28	NS	0.86	NS	0.61	NS
**Fe/U**	0.53	NS	0.45	0.00	0.63	NS	1.19	NS
**Fe/Zn**	1.22	NS	0.96	NS	1.20	NS	0.98	NS
**Mg/Mn**	0.40	NS	0.60	NS	0.95	NS	2.39	0.00
**Mg/Mo**	1.21	NS	0.84	NS	1.31	NS	1.08	NS
**Mg/Ni**	1.03	NS	0.74	NS	1.00	NS	0.98	NS
**Mg/Pb**	0.72	NS	0.09	NS	0.33	NS	0.46	NS
**Mg/Tl**	0.60	NS	0.56	0.00	0.85	NS	1.43	NS
**Mg/U**	0.40	0.00	0.51	0.00	0.56	NS	1.40	NS
**Mg/W**	0.53	NS	0.66	NS	0.65	NS	1.22	NS
**Mg/Zn**	0.93	NS	1.14	NS	1.15	NS	1.23	NS
**Mn/Ni**	3.12	NS	1.52	NS	1.32	NS	0.42	NS

Table S7. *Cont.*

Ratios	COPD-vs/COPD-mo		COPD-vs/COPD-se		COPD-vs/COPD-mi		COPD-mo/COPD-mi	
	FC	p	FC	p	FC	p	FC	p
**Mn/Pb**	3.24	NS	0.27	NS	0.48	NS	0.15	NS
**Mn/Sb**	3.13	NS	3.16	0.00	1.12	NS	0.36	NS
**Mn/Se**	4.37	NS	2.71	NS	1.84	NS	0.42	NS
**Mn/Tl**	2.44	NS	1.41	NS	1.36	NS	0.56	NS
**Mn/U**	1.44	NS	1.15	NS	0.93	NS	0.64	NS
**Mn/Zn**	4.45	NS	3.42	NS	2.01	NS	0.45	NS
**Mo/Ni**	1.52	NS	0.68	NS	1.19	NS	0.78	NS
**Mo/Pb**	0.42	NS	0.04	NS	0.36	NS	0.85	NS
**Mo/Tl**	0.60	NS	0.42	0.01	0.85	NS	1.40	NS
**Mo/U**	0.54	NS	0.46	NS	0.64	NS	1.20	NS
**Ni/Mo**	1.37	NS	1.39	NS	1.38	NS	1.01	NS
**Ni/Sb**	0.79	NS	1.92	0.01	0.84	NS	1.05	NS
**Ni/Se**	1.05	NS	1.55	NS	1.11	NS	1.06	NS
**Ni/Tl**	0.55	NS	0.85	NS	0.91	NS	1.67	NS
**Ni/U**	0.41	0.00	0.74	NS	0.55	NS	1.34	NS
**Ni/W**	0.52	NS	0.95	NS	0.60	NS	1.15	NS
**Ni/Zn**	1.08	NS	1.90	NS	0.20	NS	0.18	NS
**Pb/Ni**	1.94	NS	3.22	NS	1.68	NS	0.87	NS
**Pb/Sb**	1.80	NS	7.14	0.00	1.19	NS	0.66	NS
**Pb/Se**	2.17	NS	4.56	NS	1.75	NS	0.80	NS
**Pb/Tl**	1.40	NS	2.01	NS	1.17	NS	0.83	NS
**Pb/U**	0.46	NS	1.63	NS	0.65	NS	1.41	NS
**Pb/V**	1.31	NS	2.27	NS	0.71	NS	0.54	NS
**Pb/W**	0.53	NS	1.55	NS	0.74	NS	1.40	NS
**Pb/Zn**	1.97	NS	5.76	NS	1.88	NS	0.95	NS
**Sb/Ni**	1.18	NS	1.30	NS	1.31	NS	1.11	NS
**Sb/Pb**	1.18	NS	0.15	NS	0.51	NS	0.43	NS
**Sb/Se**	1.35	NS	1.60	NS	1.38	NS	1.03	NS

Table S7. *Cont.*

Ratios	COPD-vs/COPD-mo		COPD-vs/COPD-se		COPD-vs/COPD-mi		COPD-mo/COPD-mi	
	FC	p	FC	p	FC	p	FC	p
**Sb/Tl**	0.80	NS	0.91	NS	1.10	NS	1.38	NS
**Sb/U**	0.45	NS	0.83	NS	0.67	NS	1.49	NS
**Sb/W**	0.52	NS	0.83	NS	0.78	NS	1.51	NS
**Sb/Zn**	1.25	NS	1.89	NS	1.59	NS	1.27	NS
**Se/Tl**	0.60	NS	0.57	0.00	0.90	NS	1.51	NS
**Se/U**	0.40	0.00	0.52	0.00	0.58	NS	1.46	NS
**Se/W**	0.51	NS	0.72	NS	0.69	NS	1.35	NS
**Tl/U**	0.48	NS	0.84	NS	0.58	NS	1.20	NS
**Tl/Zn**	1.56	NS	2.32	0.01	0.21	NS	0.14	NS
**U/V**	1.01	NS	3.53	NS	1.28	NS	1.28	NS
**U/W**	1.55	NS	1.47	NS	1.10	NS	0.71	NS
**W/Zn**	0.92	NS	2.11	NS	0.68	NS	0.74	NS

.

Table S8. Metal ratios with AUC values of ROC curves higher than 0.75.

Ratios	AUC values				
	LC/HC	COPD/HC	COPD-LC /HC	COPD-s/HC	COPD-vs/HC
**Al/As**	**0.80**	<0.75	<0.75	<0.75	<0.75
**Al/Co**	**0.81**	<0.75	<0.75	<0.75	<0.75
**Al/Cr**	**0.75**	<0.75	<0.75	<0.75	<0.75
**Al/Cu**	**0.84**	<0.75	**0.83**	<0.75	<0.75
**Al/Fe**	**0.77**	<0.75	<0.75	<0.75	<0.75
**Al/Mg**	**0.86**	<0.75	<0.75	<0.75	<0.75
**Al/Mn**	**0.79**	<0.75	<0.75	<0.75	<0.75
**Al/Mo**	**0.75**	<0.75	<0.75	<0.75	<0.75
**Al/Pb**	**0.76**	<0.75	**0.82**	<0.75	<0.75
**Al/Sb**	**0.87**	<0.75	<0.75	<0.75	<0.75
**Al/Se**	**0.82**	<0.75	<0.75	<0.75	<0.75
**Al/Tl**	**0.78**	<0.75	<0.75	<0.75	<0.75
**Al/U**	**0.77**	<0.75	<0.75	<0.75	<0.75
**Al/Zn**	**0.84**	<0.75	<0.75	<0.75	<0.75
**As/Cr**	<0.75	<0.75	**0.77**	<0.75	<0.75
**As/Cu**	**0.81**	<0.75	<0.75	**0.77**	<0.75
**As/Fe**	<0.75	<0.75	**0.80**	<0.75	<0.75
**As/Mg**	<0.75	<0.75	**0.83**	<0.75	<0.75
**As/Ni**	<0.75	<0.75	<0.75	<0.75	**0.78**
**As/Sb**	**0.84**	<0.75	<0.75	<0.75	<0.75
**As/Se**	**0.78**	<0.75	**0.80**	<0.75	<0.75
**As/Tl**	<0.75	<0.75	**0.76**	<0.75	<0.75
**As/V**	<0.75	<0.75	**0.80**	**0.77**	<0.75
**As/Zn**	**0.82**	<0.75	**0.80**	<0.75	<0.75
**Cd/Co**	<0.75	<0.75	**0.80**	<0.75	<0.75
**Cd/Cr**	<0.75	<0.75	**0.82**	<0.75	<0.75
**Cd/Cu**	<0.75	<0.75	**0.82**	**0.80**	<0.75
**Cd/Fe**	<0.75	<0.75	**0.83**	**0.77**	<0.75
**Cd/Mg**	<0.75	<0.75	**0.83**	<0.75	<0.75
**Cd/Ni**	<0.75	<0.75	**0.80**	<0.75	<0.75
**Cd/Pb**	<0.75	<0.75	**0.86**	<0.75	<0.75
**Cd/Sb**	<0.75	<0.75	**0.78**	<0.75	<0.75
**Cd/Se**	<0.75	<0.75	**0.83**	<0.75	<0.75
**Cd/Tl**	<0.75	<0.75	**0.80**	<0.75	<0.75
**Cd/U**	<0.75	<0.75	**0.80**	<0.75	<0.75
**Cd/W**	<0.75	<0.75	**0.77**	<0.75	<0.75
**Cd/Zn**	<0.75	<0.75	**0.84**	**0.79**	<0.75
**Co/Cu**	<0.75	<0.75	<0.75	<0.75	**0.77**
**Co/Mn**	<0.75	<0.75	**0.76**	<0.75	<0.75
**Co/Pb**	<0.75	<0.75	**0.91**	<0.75	<0.75
**Cr/As**	<0.75	<0.75	**0.82**	<0.75	<0.75
**Cr/Mn**	<0.75	<0.75	**0.83**	<0.75	<0.75
**Cr/Ni**	<0.75	<0.75	<0.75	**0.77**	<0.75
**Cr/Sb**	**0.78**	<0.75	<0.75	<0.75	<0.75
**Cu/Al**	<0.75	<0.75	<0.75	<0.75	**0.81**
**Cu/As**	**0.76**	<0.75	**0.87**	**0.81**	<0.75
**Cu/Mn**	**0.78**	<0.75	**0.87**	<0.75	<0.75
**Cu/Mo**	<0.75	<0.75	<0.75	<0.75	**0.76**
**Cu/Ni**	<0.75	<0.75	<0.75	**0.91**	**0.76**
**Cu/Pb**	<0.75	<0.75	**0.87**	<0.75	<0.75
**Cu/Tl**	**0.77**	<0.75	<0.75	**0.79**	<0.75
**Cu/U**	<0.75	<0.75	<0.75	**0.79**	<0.75
**Fe/As**	<0.75	<0.75	**0.84**	**0.80**	<0.75
**Fe/Mn**	<0.75	<0.75	**0.85**	**0.81**	<0.75

Table S8. *Cont.*

Ratios	AUC values				
	LC/HC	COPD/HC	COPD-LC/HC	COPD-s/HC	COPD-vs/HC
**Fe/Ni**	<0.75	<0.75	<0.75	**0.85**	<0.75
**Fe/U**	<0.75	<0.75	<0.75	**0.79**	<0.75
**Mg/Al**	<0.75	<0.75	<0.75	<0.75	**0.77**
**Mg/As**	<0.75	<0.75	**0.90**	**0.83**	<0.75
**Mg/Co**	<0.75	<0.75	**0.80**	<0.75	**0.79**
**Mg/Cr**	<0.75	<0.75	**0.87**	**0.88**	<0.75
**Mg/Mn**	<0.75	<0.75	**0.92**	**0.85**	<0.75
**Mg/Ni**	<0.75	<0.75	<0.75	**0.91**	<0.75
**Mg/Pb**	<0.75	<0.75	**0.86**	<0.75	<0.75
**Mg/Tl**	<0.75	<0.75	**0.83**	**0.91**	<0.75
**Mg/U**	<0.75	<0.75	**0.80**	**0.77**	<0.75
**Mg/Zn**	<0.75	<0.75	<0.75	<0.75	**0.77**
**Mn/Co**	<0.75	<0.75	**0.76**	<0.75	<0.75
**Mn/Cr**	<0.75	**0.76**	**0.83**	<0.75	<0.75
**Mn/Cu**	**0.78**	<0.75	**0.87**	**0.76**	<0.75
**Mn/Fe**	<0.75	<0.75	**0.85**	**0.81**	<0.75
**Mn/Mg**	<0.75	<0.75	**0.90**	**0.82**	<0.75
**Mn/Ni**	<0.75	<0.75	**0.82**	<0.75	<0.75
**Mn/Pb**	<0.75	<0.75	**0.88**	<0.75	<0.75
**Mn/Sb**	**0.84**	<0.75	<0.75	**0.77**	<0.75
**Mn/Se**	<0.75	<0.75	**0.94**	**0.78**	<0.75
**Mn/Zn**	**0.81**	<0.75	**0.97**	**0.80**	<0.75
**Mo/As**	<0.75	<0.75	<0.75	**0.78**	<0.75
**Mo/Pb**	<0.75	<0.75	**0.84**	<0.75	<0.75
**Ni/As**	<0.75	<0.75	**0.86**	<0.75	<0.75
**Ni/Co**	<0.75	<0.75	**0.83**	<0.75	<0.75
**Ni/Cr**	<0.75	<0.75	**0.90**	<0.75	<0.75
**Ni/Cu**	<0.75	<0.75	**0.80**	<0.75	<0.75
**Ni/Fe**	<0.75	<0.75	**0.76**	<0.75	<0.75
**Ni/Mg**	<0.75	<0.75	**0.78**	<0.75	<0.75
**Ni/Mn**	<0.75	<0.75	**0.98**	<0.75	<0.75
**Ni/Mo**	<0.75	<0.75	**0.76**	<0.75	<0.75
**Ni/Sb**	<0.75	<0.75	**0.88**	**0.77**	<0.75
**Ni/Se**	<0.75	<0.75	**0.77**	<0.75	<0.75
**Ni/Tl**	<0.75	<0.75	**0.92**	<0.75	<0.75
**Ni/U**	<0.75	<0.75	**0.91**	<0.75	<0.75
**Ni/W**	<0.75	<0.75	**0.78**	<0.75	<0.75
**Pb/Al**	<0.75	<0.75	**0.82**	<0.75	<0.75
**Pb/As**	**0.76**	<0.75	**0.89**	<0.75	<0.75
**Pb/Co**	**0.82**	<0.75	<0.75	<0.75	<0.75
**Pb/Cr**	**0.80**	<0.75	**0.90**	<0.75	<0.75
**Pb/Cu**	**0.81**	<0.75	**0.91**	<0.75	<0.75
**Pb/Fe**	<0.75	<0.75	**0.91**	**0.77**	<0.75
**Pb/Mg**	**0.81**	<0.75	**0.90**	<0.75	<0.75
**Pb/Mn**	**0.79**	<0.75	**0.90**	<0.75	<0.75
**Pb/Mo**	**0.76**	<0.75	**0.84**	<0.75	<0.75
**Pb/Ni**	<0.75	<0.75	**0.83**	<0.75	<0.75
**Pb/Sb**	**0.90**	<0.75	**0.82**	<0.75	<0.75
**Pb/Se**	**0.88**	<0.75	**0.90**	<0.75	<0.75
**Pb/Tl**	**0.78**	<0.75	**0.90**	<0.75	<0.75
**Pb/U**	**0.76**	<0.75	**0.85**	<0.75	<0.75
**Pb/V**	<0.75	<0.75	**0.90**	<0.75	<0.75
**Pb/W**	<0.75	<0.75	**0.82**	<0.75	<0.75
**Pb/Zn**	**0.84**	<0.75	**0.91**	<0.75	<0.75

Table S8. *Cont.*

**Ratios**	**AUC values**				
	**LC/HC**	**COPD/HC**	**COPD-LC/HC**	**COPD-s/HC**	**COPD-vs/HC**
**Sb/Al**	<0.75	<0.75	<0.75	<0.75	**0.75**
**Sb/As**	<0.75	<0.75	**0.90**	<0.75	<0.75
**Sb/Co**	<0.75	<0.75	<0.75	<0.75	**0.75**
**Sb/Cr**	<0.75	<0.75	<0.75	<0.75	**0.78**
**Sb/Mn**	<0.75	<0.75	**0.91**	<0.75	<0.75
**Sb/Ni**	<0.75	<0.75	<0.75	<0.75	**0.83**
**Sb/Pb**	<0.75	<0.75	**0.78**	<0.75	**0.75**
**Se/As**	<0.75	<0.75	**0.84**	<0.75	<0.75
**Se/Cr**	**0.77**	<0.75	**0.81**	<0.75	<0.75
**Se/Mn**	<0.75	<0.75	**0.94**	**0.78**	<0.75
**Se/Ni**	**0.77**	<0.75	<0.75	<0.75	<0.75
**Se/Pb**	<0.75	<0.75	**0.88**	<0.75	<0.75
**Se/Tl**	<0.75	<0.75	**0.76**	**0.79**	<0.75
**Se/U**	<0.75	<0.75	**0.80**	<0.75	<0.75
**Tl/Al**	<0.75	<0.75	<0.75	<0.75	**0.86**
**Tl/As**	<0.75	<0.75	**0.81**	<0.75	<0.75
**Tl/Cu**	**0.77**	<0.75	<0.75	**0.79**	<0.75
**Tl/Mo**	<0.75	<0.75	<0.75	<0.75	**0.76**
**Tl/Ni**	<0.75	<0.75	**0.76**	<0.75	<0.75
**Tl/Pb**	<0.75	<0.75	**0.88**	<0.75	<0.75
**Tl/Sb**	**0.77**	<0.75	<0.75	<0.75	<0.75
**Tl/Se**	<0.75	<0.75	**0.76**	**0.79**	<0.75
**Tl/Zn**	**0.77**	<0.75	<0.75	<0.75	<0.75
**U/Al**	<0.75	<0.75	<0.75	<0.75	**0.91**
**U/As**	<0.75	<0.75	**0.82**	<0.75	**0.76**
**U/Co**	<0.75	<0.75	<0.75	<0.75	**0.79**
**U/Mn**	<0.75	<0.75	**0.76**	<0.75	<0.75
**U/Mo**	<0.75	<0.75	<0.75	<0.75	**0.81**
**U/Ni**	<0.75	<0.75	**0.75**	<0.75	**0.76**
**U/Pb**	<0.75	<0.75	**0.82**	<0.75	**0.79**
**V/Cd**	<0.75	<0.75	**0.78**	<0.75	<0.75
**V/Ni**	**0.76**	<0.75	<0.75	<0.75	<0.75
**V/Pb**	<0.75	<0.75	**0.90**	<0.75	<0.75
**V/Zn**	<0.75	<0.75	**0.80**	<0.75	<0.75
**W/Al**	<0.75	<0.75	<0.75	<0.75	**0.90**
**W/As**	<0.75	<0.75	**0.87**	**0.77**	**0.79**
**W/Mn**	<0.75	<0.75	**0.84**	<0.75	<0.75
**W/Ni**	<0.75	<0.75	<0.75	<0.75	**0.76**
**W/Pb**	<0.75	<0.75	**0.78**	<0.75	**0.84**
**W/U**	<0.75	<0.75	**0.75**	<0.75	<0.75
**W/Zn**	<0.75	<0.75	<0.75	<0.75	**0.76**
**Zn/As**	**0.78**	<0.75	**0.84**	<0.75	<0.75
**Zn/Co**	<0.75	<0.75	**0.82**	<0.75	<0.75
**Zn/Cr**	<0.75	<0.75	**0.78**	<0.75	<0.75
**Zn/Mg**	<0.75	<0.75	<0.75	<0.75	**0.75**
**Zn/Mn**	**0.81**	<0.75	**0.97**	**0.80**	<0.75
**Zn/Ni**	<0.75	<0.75	<0.75	**0.88**	<0.75
**Zn/Sb**	<0.75	<0.75	**0.80**	<0.75	<0.75
**Zn/Tl**	**0.77**	<0.75	<0.75	<0.75	<0.75
**Zn/U**	<0.75	<0.75	**0.83**	<0.75	<0.75

Table S9. Metal-metal Spearman correlations in the studied groups. HC: Healthy control; LC: lung cancer; COPD: chronic obstructively pulmonary disease; COPD-LC: patients who developed LC during the follow-up

Elements		rho	p-level
**HC**			
**Pb**	**Al**	0.87	0.00
**Se**	**Zn**	0.69	0.00
**Pb**	**Cd**	0.69	0.00
**Sb**	**Mg**	0.66	0.00
**Zn**	**Cu**	0.66	0.00
**Zn**	**Fe**	0.65	0.00
**Ni**	**Al**	0.63	0.00
**Zn**	**Co**	0.60	0.00
**Se**	**Mg**	0.59	0.00
**U**	**Tl**	0.56	0.00
**Zn**	**Mg**	0.56	0.00
**Pb**	**Ni**	0.55	0.00
**Cu**	**Fe**	0.55	0.00
**Sb**	**Se**	0.55	0.00
**As**	**Al**	0.54	0.00
**Cu**	**Co**	0.54	0.00
**Sb**	**V**	0.54	0.00
**Mo**	**Mg**	0.54	0.00
**Cr**	**Al**	0.52	0.00
**Pb**	**Cr**	0.52	0.00
**Ni**	**Co**	-0.51	0.00
**Zn**	**Ni**	-0.53	0.00
**Cu**	**Ni**	-0.55	0.00
**LC**			
**Zn**	**Co**	0.66	0.00
**Mn**	**Co**	0.66	0.00
**Cu**	**Mg**	0.65	0.00
**Cr**	**Ni**	0.64	0.00
**Cd**	**As**	0.57	0.00
**Sb**	**Cu**	0.57	0.00
**Mn**	**V**	0.56	0.00
**Sb**	**Mo**	0.56	0.00
**Mo**	**Cu**	0.55	0.00
**Cd**	**V**	0.55	0.00
**Zn**	**Cu**	0.55	0.00
**Zn**	**Mn**	0.54	0.00
**W**	**Ni**	0.53	0.00
**Pb**	**Sb**	0.53	0.00
**Cd**	**Se**	0.53	0.00
**Cu**	**Co**	0.52	0.00
**Cr**	**Mg**	-0.51	0.00
**Ni**	**Mn**	-0.54	0.00
**Se**	**Al**	-0.54	0.00
**Cr**	**Co**	-0.55	0.00
**Cu**	**Ni**	-0.55	0.00
**V**	**Al**	-0.55	0.00
**Mo**	**Cr**	-0.57	0.00
**As**	**Mg**	-0.58	0.00
**Zn**	**Ni**	-0.64	0.00
**Ni**	**Co**	-0.73	0.00

Table S9. *Cont.*

Elements		rho	p-level
**COPD**			
**Se**	**Zn**	0.73	0.00
**As**	**Al**	0.70	0.00
**Cu**	**Mg**	0.69	0.00
**Se**	**Mg**	0.67	0.00
**Cd**	**Al**	0.67	0.00
**Zn**	**Mg**	0.65	0.00
**Se**	**Cu**	0.61	0.00
**Zn**	**Cu**	0.54	0.00
**V**	**Al**	0.52	0.00
**Zn**	**Co**	0.51	0.00
**COPD-LC**			
**As**	**Mn**	0.85	0.00
**Zn**	**Mn**	0.76	0.00
**Sb**	**Co**	0.74	0.01
**W**	**Cu**	0.73	0.01
**Sb**	**Zn**	0.71	0.01
**Cu**	**V**	0.68	0.01
**Sb**	**Mo**	0.68	0.02
**W**	**V**	0.67	0.02
**Zn**	**V**	0.66	0.02
**U**	**Mo**	0.66	0.02
**Mn**	**V**	0.63	0.03
**Sb**	**Ni**	0.60	0.04
**Zn**	**Ni**	0.58	0.05
**Cr**	**Al**	-0.62	0.03
**W**	**Cd**	-0.63	0.03
**Tl**	**Se**	-0.69	0.01
**Cd**	**Cu**	-0.69	0.01

Table S10. Limit of detection (LOD) and quantification (LOQ) for elements. Limits of detection (LODs) and quantification (LOQs) were calculated with the data generated in the linearity studies as LOD = a + 3 Sy/x and LOQ = a + 10 Sy/x, where "a" is the origin ordinate and "Sy/x" the random errors in the values for the slope and intercept.

Elements (ng g^{-1})	LOD	LOQ
Mg	0.08	0.27
Al	0.06	0.20
V	0.02	0.68
Mn	0.04	0.13
Fe	0.05	0.17
Co	0.05	0.17
Ni	0.04	0.13
Cu	0.05	0.17
Zn	0.07	0.23
Cr	0.04	0.13
As	0.03	0.10
Mo	0.04	0.13
Se	0.03	0.10
Cd	0.01	0.33
Sb	0.04	0.13
W	0.04	0.13
Tl	0.02	0.68
Pb	0.04	0.13
U	0.02	0.68

Table S11. Validation of multielemental analysis by ICP-QQQ-MS using de human serum Seronorm™ Trace Elements Serum L-2 and ClinChek serum control Level II.*Additional approximate values.

Elements (n= 10)	ClinChek serum control level II (ng g-1)			Seronorm™ Trace Elements Serum L-2 (ng g-1)		
	Average	RSD (%)	Certified range	Average	RSD (%)	Certified range
Mg	23818.37	6.13	19400-23700	38993.85	4.10	28500-42800
Al	43.84	5.08	43-65	78.92	7.12	72-108
V	6.91	4.37	6.47-9.71	0.57	7.07	0.52*
Mn	5269.60	8.66	4830-7240	13.04	5.45	11.3-17
Fe	1218.55	7.88	1190-1780	1772.71	6.80	1650-2490
Co	4.92	7.22	4.59-6.89	2.59	6.70	2.15-3.24
Ni	6.13	6.80	4.77-7.15	10.77	1.41	7.6-11.4
Cu	1136.70	6.96	1150-1560	1689.25	5.24	1737-2613
Zn	1522.34	10.29	1280-1740	1687.49	9.99	1670-2510
Cr	5.67	9.42	4.53-6.79	3.64	11.19	3.12-4.69
As	15.45	7.07	14.5-24.1	1.28	8.35	0.38*
Mo	5.81	8.63	4.33-6.50	1.15	5.53	0.91*
Se	95.13	4.58	89.8-135	126.40	5.47	111-167
Cd	5.37	7.06	4.04-6.73	0.17	11.29	0.122*
Sb	5.86	1.37	5.64-8.46	15.26	8.03	16.4*
W	0.40	1.34	No certified	6.45	3.46	No certified
Tl	5.86	7.37	5.59-8.38	0.27	13.07	No certified
Pb	1.12	5.51	No certified	1.65	6.96	1.29*
U	1.17	4.87	No certified	1.86	0.29	0.53*

Table S12. Operational Conditions of ICP-QQQ-MS

**Operational Conditions**	
Nebulizer	Savillex Cross Flow 400
Sampling and skimmer cones	Nickel
RF Power	1500 W
Plasma gas flow rate	15 L min^{-1}
Carrier gas flow rate	0.65 L min^{-1}
Auxiliary gas flow	1 L min^{-1}
Sampling depth	10 mm
He flow	4.5 ml min^{-1}
H_2 flow	2 mil min^{-1}
O_2 flow	40%
Dwell time	0.3 per isotope
Torch	Shield (with long life platinum shield plate)
OctP Bias	-18.0 V
OctP RF	190 V
Energy Discrimination	-7.0 V
Isotopes monitored	^{24}Mg, ^{27}Al, ^{51}V, ^{45}Sc , ^{55}Mn, ^{57}Fe, ^{59}Co, ^{53}Cr, ^{60}Ni, ^{63}Cu, ^{65}Cu, ^{64}Zn, ^{66}Zn, ^{75}As, ^{78}Se, ^{80}Se, ^{95}Mo,^{103}Rh, ^{112}Cd, ^{114}Cd, ^{182}W, ^{205}Tl, ^{208}Pb, ^{209}Bi and ^{238}U
Replicates	5

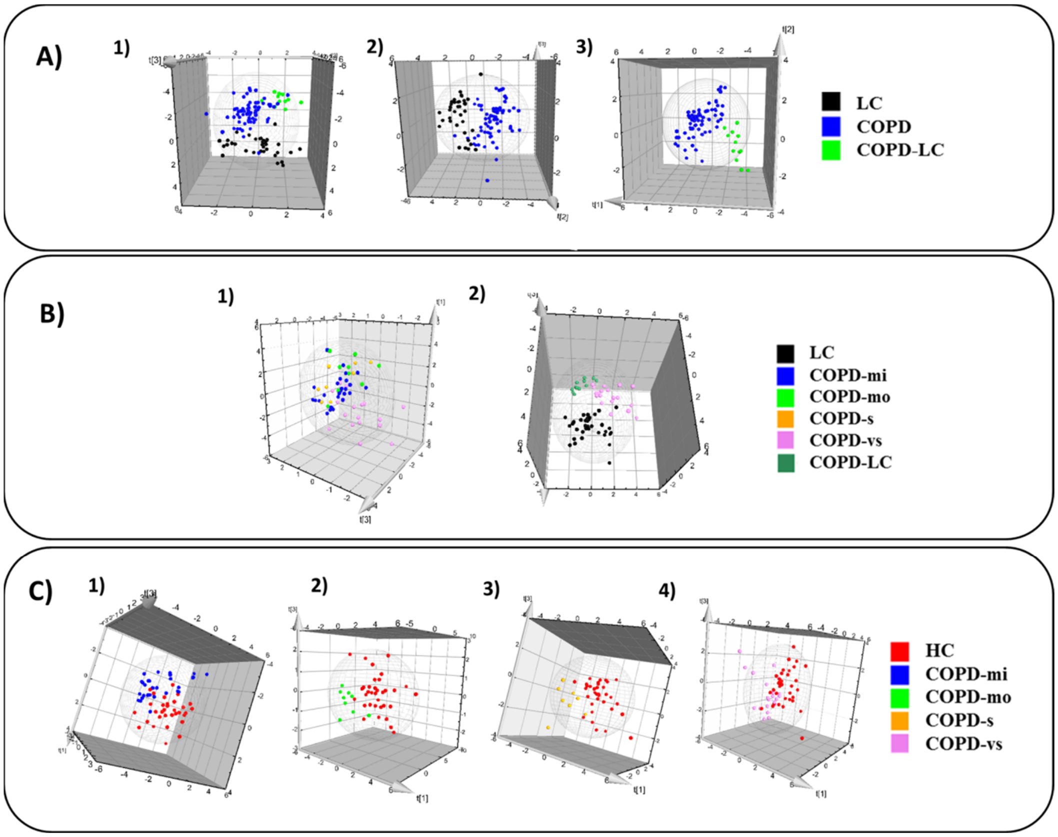

Figure S1. PLS-DA plots of metal human serum comparison between (**A**) COPD, LC and COPD-LC groups (1); LC and COPD groups (2) and COPD and COPD-LC groups, (**B**) COPD-mi, COPD-mo, COPD-s and COPD-vs (1) and COPD-vs, LC and COPD-LC groups (2), (**C**) HC and COPD-mi (1), HC and COPD-mo(2), HC and COPD-s (3), HC and COPD-vs (4) groups. HC: Healthy control; LC: lung cancer; COPD: chronic obstructively pulmonary disease; COPD-LC: patients who developed LC during the follow-up; COPD-vs: very severe COPD; COPD-s: severe COPD, COPD-mo: moderate COPD and COPD-mi: mild COPD.

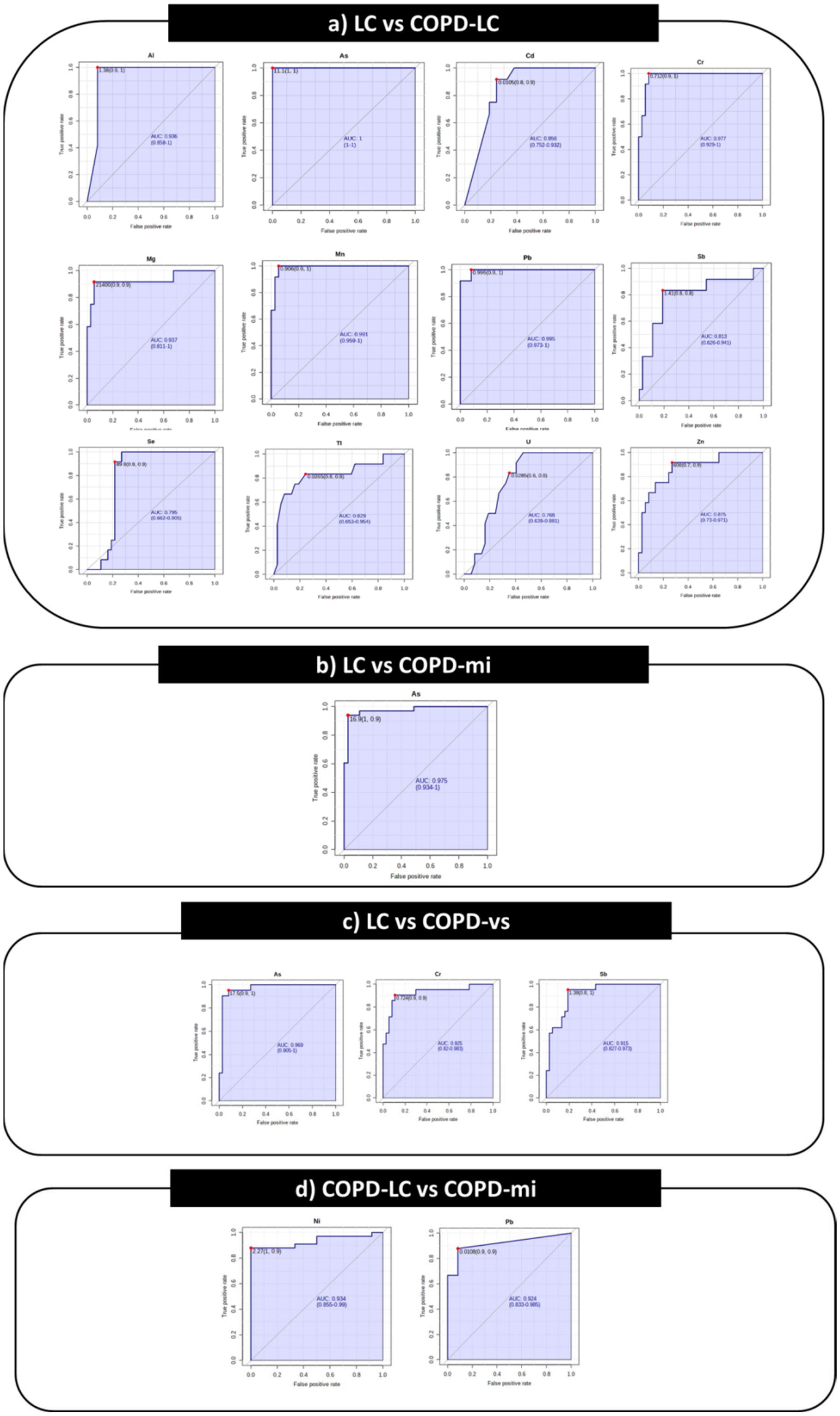

Figure S2. ROC curves corresponding to the elements that presented significant differences between (**a**) LC and COPD-LC groups, (**b**) LC and COPD-mi, (**c**) LC and COPD-vs and d) COPD-LC and COPD-mi considering good values of specificity and specificity AUC values higher than 0.75. The rest of the comparison showed AUC values < 0.75.
